# Tumor microenvironment defines the invasive phenotype of *AIP*-mutation-positive pituitary tumors

**DOI:** 10.1038/s41388-019-0779-5

**Published:** 2019-03-12

**Authors:** Sayka Barry, Eivind Carlsen, Pedro Marques, Craig E. Stiles, Emanuela Gadaleta, Dan M. Berney, Federico Roncaroli, Claude Chelala, Antonia Solomou, Maria Herincs, Francisca Caimari, Ashley B. Grossman, Tatjana Crnogorac-Jurcevic, Oliver Haworth, Carles Gaston-Massuet, Márta Korbonits

**Affiliations:** 1 Centre for Endocrinology, William Harvey Research Institute, Barts and The London School of Medicine, Queen Mary University of London, London, EC1M 6BQ UK; 2 Department of Pathology, STHF, N-3710 Skien, Norway; 3 Molecular Oncology, Barts Cancer Institute, Barts and The London School of Medicine, Queen Mary University of London, London, EC1M 6BQ UK; 4 Division of Neuroscience & Experimental Psychology, University of Manchester, Manchester, M13 9PL UK

**Keywords:** Extracellular signalling molecules, Prognostic markers

## Abstract

The molecular mechanisms leading to aryl hydrocarbon receptor interacting protein (*AIP*) mutation-induced aggressive, young-onset growth hormone-secreting pituitary tumors are not fully understood. In this study, we have identified that *AIP*-mutation-positive tumors are infiltrated by a large number of macrophages compared to sporadic tumors. Tissue from pituitary-specific *Aip*-knockout (*Aip*^*Flox/Flox*^*;Hesx1*^*Cre/+*^) mice recapitulated this phenotype. Our human pituitary tumor transcriptome data revealed the “epithelial-to-mesenchymal transition (EMT) pathway” as one of the most significantly altered pathways in *AIP*pos tumors. Our in vitro data suggest that bone marrow-derived macrophage-conditioned media induces more prominent EMT-like phenotype and enhanced migratory and invasive properties in *Aip*-knockdown somatomammotroph cells compared to non-targeting controls. We identified that tumor-derived cytokine CCL5 is upregulated in *AIP*-mutation-positive human adenomas. *Aip*-knockdown GH3 cell-conditioned media increases macrophage migration, which is inhibited by the CCL5/CCR5 antagonist maraviroc. Our results suggest that a crosstalk between the tumor and its microenvironment plays a key role in the invasive nature of *AIP*-mutation-positive tumors and the CCL5/CCR5 pathway is a novel potential therapeutic target.

## Introduction

Heterozygous mutations in the aryl hydrocarbon receptor interacting protein (*AIP*) gene are present in about fifth of both familial isolated pituitary adenoma and childhood-onset sporadic somatotroph adenomas [1]. Patients with germline *AIP* mutations (*AIP*pos) have distinct clinical features, such as young age at diagnosis, large, invasive, sparsely-granulated adenomas with poor response to somatostatin analogs [1–6]. Identification of factors and molecular pathways leading to this aggressive phenotype are of particular importance to predict tumor behavior and identify novel therapeutic targets.

Crosstalk between tumor cells and components of the tumor microenvironment plays a key role in tumor invasion [7–10]. The tumor microenvironment includes immune cells, fibroblasts, endothelial cells, extracellular matrix, and numerous secreted soluble factors such as cytokines, altogether representing a dynamic autocrine–paracrine interaction network that influences tumor behavior. Relatively sparse data are available on the tumor microenvironment of pituitary adenomas. Previous studies found low level of macrophage [11] or lymphocyte [12] infiltration, while a more recent study showed that the presence of hematopoietic CD45+ cells was associated with poor clinical outcome [13] or invasiveness in sparsely granulated somatotroph adenomas [14]. Understanding interactions between tumor cells and the tumor microenvironment may therefore provide novel therapeutic targets.

Our observation of increased macrophage infiltration in *AIP*pos tumors compared to sporadic somatotrophinomas, combined with gene expression profiling of freshly frozen *AIP*pos samples indicating altered tumor microenvironment, prompted us to study the invasive behavior of *AIP*pos tumors in terms of the microenvironment. We found that the tumor-derived cytokine CCL5 is upregulated in *AIP*-mutation-positive human adenomas. The pituitary-specific *Aip*-knockout mouse (*Aip*^*Flox/Flox*^*;Hesx1*^*Cre/+*^) show increased macrophage content, similar to the human *AIP*pos tumors. In our in vitro experiments, supernatant of a stable *Aip*-knockdown somatomammotroph cell line stimulated macrophage migration via CCL5/CCR5 pathway, while macrophage-derived factors lead to epithelial-to-mesenchymal transition (EMT), increased migration, and invasion in pituitary somatotroph cells.

## Results

### Analysis of the components of the tumor microenvironment in *AIP*pos tumors

**Fig. 1:**
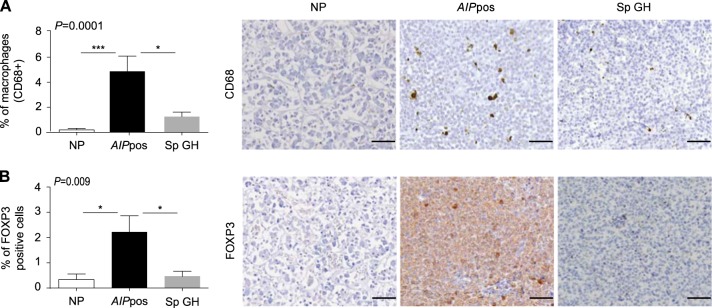
Alterations of the components of the tumor microenvironment in *AIP*pos tumors. Immunohistochemical analyses of CD68 and FOXP3 in *AIP-*mutation-positive human somatotroph adenomas (*AIP*pos), sporadic somatotroph adenomas (Sp GH), and normal pituitaries. Graphs on the left are showing the percentage of CD68 (**a**) and FOXP3 (**b**) positive cells per high-power magnification field, counted on 3–5 random fields at ×400. Plotted data were expressed as mean ± SEM. Statistical analysis was performed using Kruskal–Wallis test followed by Conover–Inman test for individual comparisons; significance between groups are marked as *, <0.05, ***, <0.001. Representative images (right panels) show more CD68 (macrophages) and FOXP3-positive (T-reg) cells in *AIP*pos tumors compared to sporadic somatotroph adenomas. Analyzed number of samples for normal pituitary, *AIP*pos tumors and sporadic GH tumors, respectively, are as follows: CD68 (*n* = 9, 9, and 17) and FOXP3 (*n* = 11, 9, and 17). All images are ×200 magnification and scale bar = 100 µm

To understand the tumor microenvironment of *AIP*pos tumors, we evaluated expression of several key components of the tumor microenvironment using specific molecular markers for macrophages (CD68), T-reg cells (FOXP3), cytotoxic T cells (CD8), and memory T cells (CD45RO). Immunostaining with CD68 showed a remarkable increase in the presence of CD68-positive cells in *AIP*pos tumors compared to sporadic adenomas (*P* = 0.01) or normal pituitaries (*P* = 0.001) (Fig. 1a). *AIP*pos tumors also expressed a significantly higher number of FOXP3+ T-reg cells compared to sporadic adenomas (*P* = 0.02) or normal tissues (*P* = 0.01) (Fig. 1b). No significant differences were found in cytotoxic (CD8) or memory T cell (CD45RO) content (Fig. S1A, B).

### EMT signatures in *AIP*pos tumors

Microarray gene expression profiling of normal pituitary and familial or sporadic growth hormone (GH)-secreting tumors (*n* = 15) identified several significantly altered pathways (Table S1). There were 3,025 differentially expressed genes for *AIP*pos vs. normal pituitaries and 1,564 differentially expressed genes for sporadic tumors vs. normal pituitaries. The most significantly altered canonical pathways are shown in Fig. S2. The “Regulation of the Epithelial-Mesenchymal Transition Pathway” was one of the most significantly altered pathways (47 genes with 16 upregulated and 31 downregulated) in *AIP*pos GH tumors compared to sporadic adenomas (Table 1). Six EMT genes (*CDH1, CTNNB1, ESRP1, EPCAM, PERP*, and *ZEB1*) were selected for further validation (Table S2).

**Table 1:** Forty-seven known EMT-related genes in *AIP*pos somatotroph adenomas

Symbol	Entrez gene name	Affymetrix	Fold change
ADAM17	ADAM metallopeptidase domain 17	205746_s_at	2.32
AKT3	v-akt murine thymoma viral oncogene homolog 3	242876_at	−2.92
APC	adenomatous polyposis coli	203527_s_at	2.80
BRAF	B-Raf proto-oncogene, serine/threonine kinase	206044_s_at	−3.90
CDH1	cadherin 1, type 1, E-cadherin (epithelial)	201131_s_at	−27.00
CDH2	cadherin 2, type 1, N-cadherin (neuronal)	203440_at	−17.74
CLDN3	claudin 3	203954_x_at	−3.14
CTNNB1	catenin (cadherin-associated protein), beta-1, 88 kDa	223679_at	−4.07
EGFR	epidermal growth factor receptor	224999_at	−5.65
EPCAM	epithelial cell adhesion molecule	201839_s_at	−2.42
ESRP1	epithelial splicing regulatory protein 1	225846_at	−32.31
ESRP2	epithelial splicing regulatory protein 2	219395_at	−5.92
FGF13	fibroblast growth factor 13	205110_s_at	−7.93
FGFR1	fibroblast growth factor receptor 1	222164_at	−4.63
FGFR2	fibroblast growth factor receptor 2	203638_s_at	−4.64
FGFR3	fibroblast growth factor receptor 3	204379_s_at	−9.55
FZD3	frizzled class receptor 3	239082_at	−7.35
FZD5	frizzled class receptor 5	221245_s_at	−3.71
FZD7	frizzled class receptor 7	203706_s_at	−15.41
GSK3B	glycogen synthase kinase 3 beta	226183_at	2.89
HGF	hepatocyte growth factor (hepapoietin A; scatter factor)	209960_at	−4.20
HRAS	Harvey rat sarcoma viral oncogene homolog	212983_at	−2.11
JAG2	jagged 2	32137_at	−2.97
JAK1	Janus kinase 1	239695_at	−4.41
LEF1	lymphoid enhancer-binding factor 1	221558_s_at	3.57
LOX	lysyl oxidase	215446_s_at	2.76
MAP2K5	mitogen-activated protein kinase kinase 5	204756_at	2.01
MMP2	matrix metallopeptidase 2 (gelatinase A, 72 kDa gelatinase, 72 kDa type IV collagenase)	201069_at	4.95
MMP9	matrix metallopeptidase 9 (gelatinase B, 92 kDa gelatinase, 92 kDa type IV collagenase)	203936_s_at	2.82
NOTCH2	notch 2	202443_x_at	−4.95
PERP	PERP, TP53 apoptosis effector	222392_x_at	−3.73
PIK3C2A	phosphatidylinositol-4-phosphate 3-kinase, catalytic subunit type 2 alpha	241905_at	−7.36
PIK3C3	phosphatidylinositol 3-kinase, catalytic subunit type 3	232086_at	3.85
PIK3CB	phosphatidylinositol-4,5-bisphosphate 3-kinase, catalytic subunit beta	217620_s_at	−2.41
PIK3CG	phosphatidylinositol-4,5-bisphosphate 3-kinase, catalytic subunit gamma	239294_at	4.24
PSENEN	presenilin enhancer gamma secretase subunit	218302_at	2.92
RELA	v-rel avian reticuloendotheliosis viral oncogene homolog A	201783_s_at	–2.10
RRAS2	related RAS viral (r-ras) oncogene homolog 2	212589_at	–4.12
SMAD2	SMAD family member 2	203076_s_at	2.50
SMAD3	SMAD family member 3	218284_at	–4.13
TCF4	transcription factor 4	212385_at	2.92
TCF7L1	transcription factor 7-like 1 (T cell specific, HMG-box)	221016_s_at	−2.41
TGFB2	transforming growth factor, beta 2	209909_s_at	8.74
TWIST1	twist family bHLH transcription factor 1	213943_at	−4.84
WNT4	wingless-type MMTV integration site family, member 4	208606_s_at	7.04
WNT5A	wingless-type MMTV integration site family, member 5A	213425_at	−4.70
ZEB1	zinc-finger E-box binding homeobox 1	210875_s_at	3.64

### Validation of EMT markers with quantitative reverse transcriptase PCR and immunohistochemistry

**Fig. 2:**
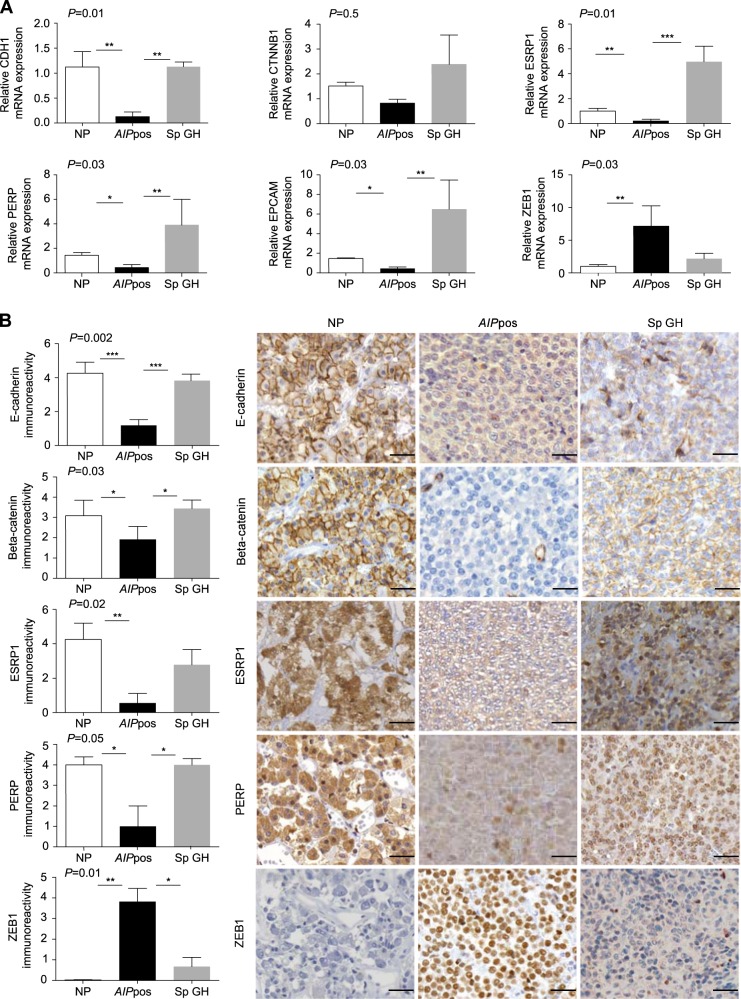
Validation of the selected EMT markers at the mRNA and protein levels. **a** RT-qPCR validation of downregulated (*CDH1, CTNNB, ESRP1, PERP*, and *EPCAM*) and upregulated (*ZEB1*) EMT genes. RNA expression of the down- and upregulated genes in *AIP*pos tumors (*n* = 6) compared to normal pituitaries (*n* = 5) and sporadic GH tumors (*n* = 3) show confirmation of the gene expression profile data. **b** Immunohistochemical analysis of downregulated (*CDH1, CTNNB, ESRP1*, and *PERP*) and upregulated (*ZEB1*) EMT genes. Protein expression in graphical form and with representative images in *AIP*pos tumors compared to normal pituitaries and sporadic GH tumors. E-cadherin: Normal pituitary cells are showing uniform strong to moderate membranous staining. *AIP*pos GH tumor displays weak diffuse cytoplasmic positivity without any membranous staining. Sporadic GH adenoma shows membranous and granular cytoplasmic positivity. Beta-catenin: Normal pituitary cells are showing strong to moderate membranous immunoreactivity. *AIP*pos GH tumor displays discontinuous cytoplasmic expression. Sporadic GH shows membranous and granular cytoplasmic positivity. ESRP1: Normal pituitary cells are showing strong cytoplasmic immunoreactivity. *AIP*pos GH tumor shows weak cytoplasmic expression. Sporadic GH tumor shows universal cytoplasmic and moderate to strong nuclear positivity. PERP: Normal pituitary cells are showing strong granular cytoplasmic positivity. *AIP*pos GH tumor shows cytoplasmic expression with the nuclear atypia. Sporadic GH tumor shows granular cytoplasmic and nuclear positivity of variable strength. ZEB1: Normal pituitary cells are completely negative. *AIP*pos GH tumor exhibits uniform moderate to strong positive nuclear staining. Sporadic GH tumor shows weak to moderate nuclear staining in the majority of the tumor cells. Overall *P* value for multiple comparison is shown in the left upper corner of each graph, while significance between groups are marked with *, <0.05, **, <0.01, ***, <0.001 (Kruskal–Wallis test followed by Conover–Inman test). All images are ×400 magnification and scale bar = 50 µm

Two-step validation using quantitative reverse transcriptase PCR (RT-qPCR) and immunohistochemistry confirmed our gene expression profiling data (Tables S3 and S4; Fig. 2a, b). E-cadherin *(CDH1*) mRNA was downregulated in *AIP*pos tumors (*P* = 0.004) compared to the normal pituitaries and sporadic GH adenomas (*P* = 0.001) (Fig. 2a). A significantly lower expression of E-cadherin was seen in *AIP*pos tumors compared to normal pituitaries (*P* = 0.0008) and to sporadic somatotrophinomas (*P* = 0.001) (Fig. 2b). No significant transcript level change was seen for *CTNNB1* (cadherin-associated protein, beta-1) coding for β-catenin (Fig. 2a); however, there was a significant difference at the protein level. Normal pituitary showed strong homogeneous membranous β-catenin staining, whereas absent or weak granular membranous beta-catenin expression was observed in 40% of sporadic tumors and 83% of *AIP*pos tumors (*AIP*pos vs. normal pituitary *P* = 0.01, *AIP*pos vs. sporadic somatotrophinoma *P* = 0.04) (Fig. 2b). *ESRP1* (epithelial splicing regulatory protein 1), a novel molecular marker of EMT, was significantly downregulated in *AIP*pos tumors compared to the normal pituitaries (*P* = 0.005) and sporadic tumors (*P* = 0.0001) at the mRNA level (Fig. 2a). ESRP1 protein expression was significantly decreased in *AIP*pos tumors compared to normal pituitaries (*P* = 0.005) (Fig. 2b). *PERP* (TP53 apoptosis effector), an EMT-related gene, was significantly downregulated in *AIP*pos tumors both at the RNA and protein level compared to normal pituitaries (*P* = 0.01 and 0.03) and to sporadic somatotrophinomas (*P* = 0.002 and 0.02) (Fig. 2a, b). The significant transcriptional downregulation of *EPCAM* (epithelial cell adhesion molecule; CD326) was confirmed in *AIP*pos tumors compared to normal pituitaries (*P* = 0.01) and sporadic adenomas (*P* = 0.004) (Fig. 2a). Upregulation of *ZEB1* (zinc-finger E-box binding homeobox 1), one of the master regulators of EMT, had higher mRNA expression (vs. normal pituitary *P* = 0.005) and increased nuclear protein expression (vs. normal pituitary *P* = 0.006; vs. sporadic somatotrophinomas *P* = 0.01) (Fig. 2a, b).

### Macrophage secreted factors induce EMT-like phenotype and enhance migration and invasion of GH3-*Aip*-KD cells

*AIP*pos tumors contain a higher number of macrophages and show an EMT signature corresponding to recent results linking tumor-associated macrophages with EMT, which might be critical for invasive behavior [15]. Therefore, we investigated the impact of tumor-associated macrophages in the invasive behavior of *Aip*-knockdown GH3 cells using in vitro co-cultures. Freshly isolated rat bone marrow-derived macrophages (confirmed with macrophage markers CD11b and CD68) grown in Roswell Park Memorial Institute (RPMI) medium were stimulated with 320 nM phorbol myristate acetate (PMA) for 24 h. Media was replaced with RPMI which was collected at 72 h and then used as macrophage-derived conditioned medium (MCM) for the subsequent analysis (Fig. S3A and B). We used lentiviral-transduced shRNA knockdown of *Aip* in the rat pituitary somatomammotroph cell line GH3 (GH3-*Aip*-KD) that show 80% reduced AIP protein expression (Fig. S3C). In order to verify the functional effects of *Aip* knockdown, we have used two different clones with 50% and 80% level of *Aip* knockdown. Both the 50% and 80% knockdowns of *Aip* show increased proliferation and colony formation compared to non-targeting controls (GH3-NT) (Fig. S3D), as previously shown in *Aip* knockdown [4, 16] or knockout cells [17]. GH3-*Aip*-KD (80%) and GH3-NT cells were incubated with MCM. A second set of cells following 72 h MCM treatment were incubated with Dulbecco’s modified Eagle’s medium (DMEM) for a further 72 h, to study if the MCM-induced EMT-like changes could be reversed, representing the mesenchymal-to-epithelial transition process.

**Fig. 3:**
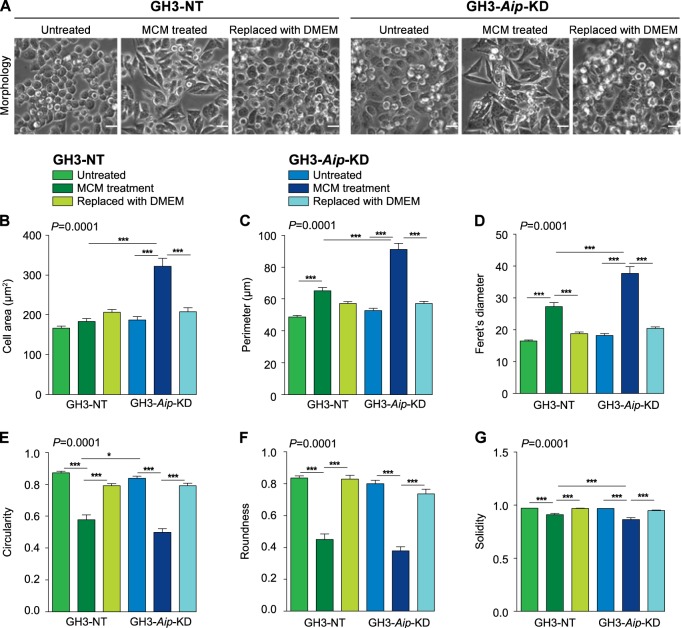
Macrophage-conditioned media induce EMT-like phenotype in GH3-*Aip*-KD cells. **a** Macrophage-conditioned media (MCM) induces an EMT-like phenotype. Morphological changes in GH3-*Aip*-KD and control GH3-NT (representative phase contrast images, top panels) and quantification of cell morphology (bottom panels). Untreated and mesenchymal-to-epithelial transition state (replaced with DMEM) GH3-*Aip*-KD and GH3-NT cells showed an epithelial cobblestone-like morphology (phase contrast images: first, third, fourth, and sixth panels) whereas MCM-treated cells both GH3-NT and GH3-*Aip*-KD become spindle shaped and show mesenchymal like morphology (phase contrast images: second and fifth panels). Untreated GH3-NT and GH3-*Aip*-KD cells show no significant differences in cell size and shape. Morphology of the cells was quantified using six different parameters (ImageJ). Around 100 cells from each condition were evaluated. There was an increase in cell area (**b**) and perimeter (**c**) in MCM-treated GH3-NT and GH3-*Aip*-KD cells, while, cells without MCM treatment showed no difference. **d** Feret’s diameter (measure of cell elongation) was significantly higher in MCM-treated cells, indicating more elongated cell shape. **e**, **f** Circularity and roundness (a value closer to one is indicate more circular/rounded cells and close to zero indicated an elongated shape): untreated and mesenchymal-to-epithelial transition state cells of GH3-NT and GH3-*Aip*-KD showed more circular and rounded shape than MCM-treated cells. **g** Solidity, defined as the ratio of cell area to the enclosing convex polygon area, indicates the stiffness and deformability of cells, was decreased in both GH3-NT and GH3-*Aip*-KD cells undergoing EMT. Overall *P* value for multiple comparison is shown in the left upper corner of the graphs, while significance between groups are marked with *, <0.05, ***, <0.001 (two-way ANOVA followed by Newman–Keuls multiple comparison test). *n* = 3, performed in triplicates on three independent days. Scale bar = 25 µm

Cell morphology analysis by ImageJ demonstrated that untreated GH3-NT and GH3-*Aip*-KD cells show no significant differences in cell size and shape (Fig. 3). MCM-treated GH3-*Aip*-KD and GH3-NT cells both underwent EMT-like changes with elongated, spindle-shape mesenchymal morphology (Fig. 3a). However, these changes were significantly more pronounced in GH3-*Aip*-KD cells as shown by cell shape analysis [18]: they have approximately 62% increased cell surface area, 40% larger perimeter and become 42% more elongated than the GH3-NT cells (Fig. 3b–d). Roundness and circularity cell parameters between MCM-treated GH3-*Aip*-KD and GH3-NT cells were not significantly different. Cell solidity or stiffness are important features of cellular plasticity [19]. Solidity index was decreased for both cell lines following MCM treatment, but GH3-*Aip*-KD cells had significantly lower solidity compared to GH3-NT cells, suggesting that these cells are more deformable (Fig. 3g). This flexibility is required for migration/invasion through the extracellular matrix and we observed this in the in vitro migration assay where cells need to traverse 8 µm pores of transwell inserts (see data below). After washing off MCM and 72 h treatment with DMEM, both cell lines reverted back to a rounded morphology, with circularity and roundness values returning back to almost one, and showing increased solidity, representing mesenchymal-to-epithelial transition (Fig. 3e–g).

**Fig. 4:**
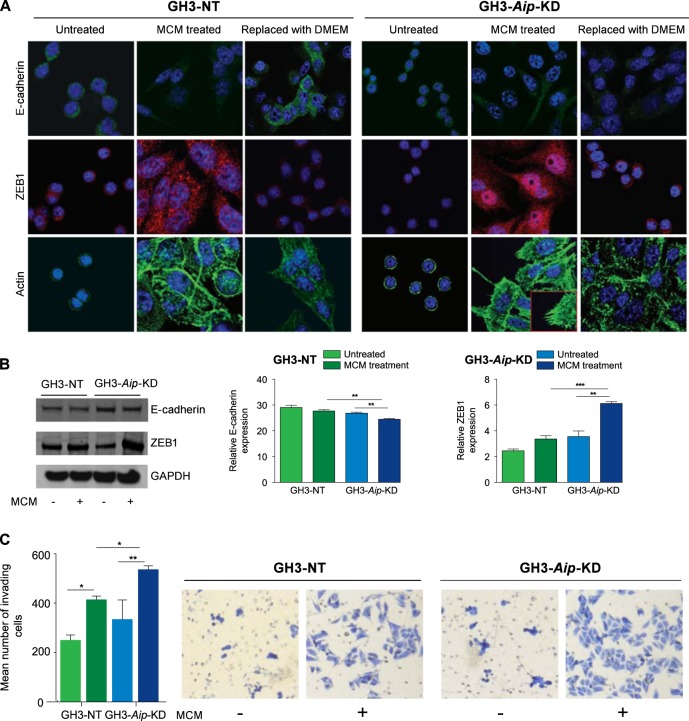
Alterations of the EMT markers in GH3-NT and GH3-*Aip*-KD cells. **a** Immunofluorescence analysis of E-cadherin, ZEB1, and actin in GH3-NT and GH3-*Aip*-KD cells with or without MCM at 72 h. Untreated cells of GH3-NT and GH3-*Aip*-KD cells show membranous localization of E-cadherin and low level of cytoplasmic ZEB1 expression, while MCM-treated cells show lack of membranous but increased cytoplasmic E-cadherin expression and a significant increase in nuclear and cytoplasmic ZEB1 expression. After removal of MCM and culturing cells in 10% DMEM for 72 h, the cells revert back to their cobblestone-like morphology (mesenchymal-to-epithelial transition state), increased E-cadherin expression and localization as well as reduced ZEB1 expression. Actin staining revealed that untreated cells show cortical rings of actin. MCM-treated GH3-NT cells show granular pattern of actin with less actin stress fibers while GH3-*Aip*-KD cells showed prominent actin stress fibers and numerous actin spikes (inset). In contrast, in mesenchymal-to-epithelial transition state cells are gradually return to the original state of their actin cytoskeleton (×63 magnifications). DAPI was used to stain the nuclei; the pictures are representative of at least three experiments. **b** Immunoblotting (densitometric analysis and representative images) suggested that E-cadherin levels were significantly decreased and ZEB1 significantly increased in MCM-treated GH3-*Aip*-KD cells compared to GH3-NT cells. Overall *P* value for multiple comparison is shown, while significance between groups are marked with **, <0.01, ***, <0.001; two-way ANOVA followed by Newman–Keuls multiple comparison test. **c** Invasion assays showing that MCM treatment increases the invasion of GH3-NT and GH3-*Aip*-KD cells. Bar charts show the mean number of invading cells through the Matrigel-coated transwell chambers measured after 72 h. Both GH3-NT and GH3-*Aip*-KD cells show significant increase in invasion compared to the untreated cells, but was more significant in GH3-*Aip*-KD cells. Representative photographs of invading cells are shown (×10), right panels. *P* values indicated *, <0.05, **, <0.01; two-way ANOVA followed by Newman–Keuls multiple comparison test. Data represent mean values of three independent experiments

Analysis of EMT markers by immunofluorescence analysis demonstrated that while untreated cells show membranous E-cadherin and little cytoplasmic ZEB1 expression, MCM-treated cells show lack of membranous E-cadherin expression and a significant increase in nuclear and cytoplasmic ZEB1 expression (Fig. 4a). Ingenuity pathway analysis of *AIP*pos tumors transcriptome identified altered actin cytoskeleton remodeling pathways. Actin staining of GH3 cells showed that untreated cells have cortical actin rings. After MCM treatment, GH3-NT cells show a granular pattern of actin with less actin stress fibers and actin spikes whereas GH3-*Aip*-KD cells showed a mesenchymal phenotype with elongated morphology with prominent actin stress fibers and numerous actin spikes (Fig. 4a). In the mesenchymal-to-epithelial transition state, cells re-organize actin cytoskeleton and reverse their morphology. Western blot analysis showed that expression of E-cadherin was significantly decreased (*P* = 0.006) whereas expression of ZEB1 was increased in MCM-treated GH3-*Aip*-KD cells (*P* = 0.001) compared to GH3-NT cells (Fig. 4b). These results suggest that macrophage-derived soluble factors could promote an EMT-like phenotype in rat pituitary GH3 cells.

Next, we assessed the functional consequences of EMT to understand if MCM treatment was altering migration and invasion capacity of GH3-*Aip*-KD and GH3-NT cells. There was no difference in baseline migration between the two cell lines. Incubation with MCM significantly increased cellular migration of GH3-*Aip*-KD cells compared to untreated cells (Fig. S4). MCM treatment increased invasion in both cell types, but more significantly in GH3-*Aip*-KD (*P* = 0.03) (Fig. 4c). These results indicate that activated macrophage-derived factors increase migration/invasion of GH3-*Aip*-KD cells while GH3-NT shows no or little response.

### The effect of tumor-derived factors on macrophage migration

**Fig. 5:**
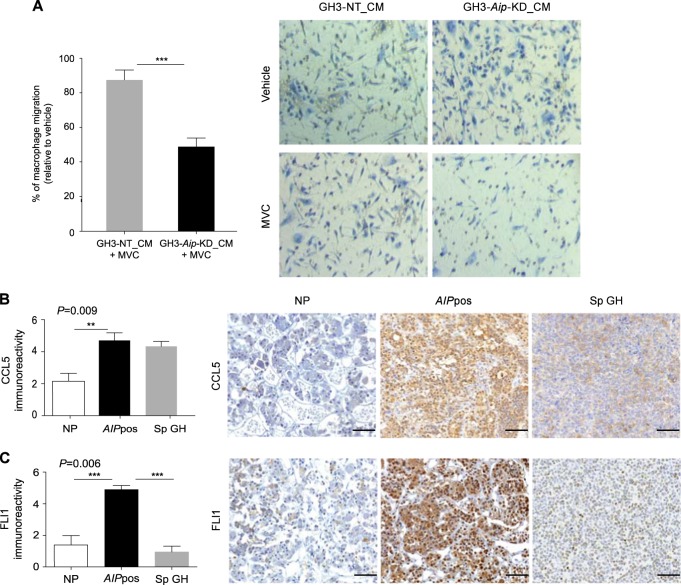
The role of the CCL5 pathway in macrophage migration. **a** Migration assays showing that macrophage migration was significantly reduced towards GH3-*Aip*-KD cell-derived conditioned media compared to GH3-NT control cell-derived conditioned media. Macrophages were in vitro treated with maraviroc (200 nM) for 24 h and the migration in response to GH3-*Aip*-KD-conditioned media and GH3-NT conditioned media was evaluated. Cells were counted in nine random fields and data are presented as mean ± SEM, *n* = 3. Graph showing the percentage of the MVC-treated migrated macrophages towards the GH3-NT and GH3-*Aip*-KD-conditioned medium relative to the vehicle. V vehicle, MVC maraviroc. *P* values indicated ***, <0.001; *t*-test. **b** Immunohistochemical analysis of CCL5 in normal pituitary (NP, *n* = 11), *AIP-*mutation-positive somatotroph adenomas (*AIP*pos GH, *n* = 12) and sporadic somatotroph adenomas (Sp GH, *n* = 17). Graph showing that CCL5 is highly upregulated in *AIP*pos tumors compared to the normal pituitary (left panel). Right panels show the representative images of CCL5 staining. *P* values indicated **, <0.01; one-way ANOVA with Bonferroni multiple comparison test. All images are ×200 magnification and scale bar = 100 µm. **c** Immunohistochemical analysis of FLI1 in normal pituitary (NP, *n* = 11), *AIP-*mutation-positive somatotroph adenomas (*AIP*pos GH, *n* = 12) and sporadic somatotroph adenomas (Sp GH, *n* = 17). Graph showing that FLI1 is highly upregulated in *AIP*pos tumors compared to the normal pituitary (left panel) and Sp GH tumors (left panel). Right panels show the representative images of FLI1 staining. *P* values indicated ***, <0.001; one-way ANOVA with Bonferroni multiple comparison test. All images are ×200 magnification and scale bar = 100 µm

As we found that macrophage-derived factors significantly altered the phenotypic and functional characteristics of GH3-*Aip*-KD cells compared to the GH3-NT cells, we tested effects of tumor-derived factors on macrophage recruitment: GH3-*Aip*-KD cell-derived conditioned medium was used as chemoattractant for migration of macrophages. Increased macrophage migration was observed towards the GH3-*Aip*-KD cell-derived medium compared to GH3-NT cell-conditioned medium (Fig. S5A). These results demonstrate that the GH3-*Aip*-KD cells release chemotactic factors that might enable increased migration towards tumor cells. Next, we explored human gene expression data to search for potential chemotactic factors in *AIP*pos tumors that could enhance macrophage migration. Our top candidate was chemokine C–C motif ligand 5 (CCL5). CCL5, also known as RANTES (*r*egulated upon *a*ctivation, *n*ormal *T* cell *e*xpressed and presumably *s*ecreted), a protein known to be involved in recruitment of macrophages [20], was significantly upregulated (~6-fold) in *AIP*pos tumors compared to normal pituitary and sporadic tumors. CCL5 is a ligand for the CCR5 receptor expressed by macrophages. We hypothesized that tumor-derived CCL5 increases macrophage migration via activating CCR5 on macrophages. To test this hypothesis first we performed macrophage chemotaxis assays using recombinant CCL5 as chemoattractant and then used the CCR5 inhibitor maraviroc, an FDA-approved drug, to block their interaction. Recombinant CCL5 increased activated macrophage migration, and this was inhibited by maraviroc (Fig. S5B). Subsequent experiments using GH3-*Aip*-KD-conditioned media showed that maraviroc also inhibits macrophage migration towards GH3-*Aip*-KD-conditioned media compared to GH3-NT-conditioned media (Fig. 5a), indicating the role of CCL5–CCR5 interaction in this phenomenon. Furthermore, immunohistochemical analysis revealed higher levels of CCL5 expression in *AIP*pos tumors than normal pituitary (*P* = 0.001, Fig. 5b) and no difference between sporadic tumors vs. normal pituitaries. CCL5 levels were elevated in GH3-*Aip*-KD condition media compared to GH3-NT conditioned media (Fig. S5B). To better understand the mechanism of macrophage recruitment via CCL5, we mined our gene expression profile data to identify potential regulators of CCL5 in *AIP*pos tumors. *FLI1* (Friend leukemia virus integration site-1), a transcription factor, was found to be four-fold upregulated in *AIP*pos tumors compared to the normal pituitary. There was a significantly increased expression of FLI1 in *AIP*pos tumors compared to either sporadic tumors (*P* = 0.003) or normal pituitaries (*P* = 0.02) (Fig. 5c), therefore explaining upregulated CCL5 [21].

### Loss of *AIP* increases macrophage infiltrates in *Aip*-knockout mice

**Fig. 6:**
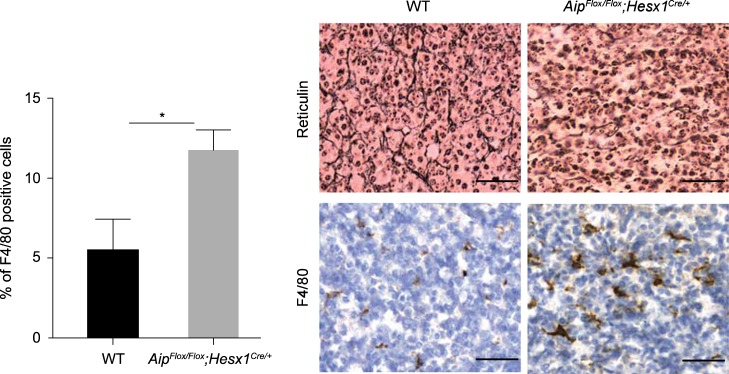
Loss of *AIP* increases macrophage infiltrates in the *Aip*-knockout mice. Reticulin staining of wild type (WT) and homozygote knockout (*Aip*^*Flox/Flox*^;*Hesx1*^*Cre/+*^*)* pituitary tissue showing disrupted reticulin network in the knockout animal. The bar graphs show the increased number of macrophages in *Aip*-knockout mice compared to the wild type. Representative images of macrophage infiltration in wild type and homozygote *Aip*-knockout mice as determined by F4/80 staining and quantified as the percentage of F4/80+ cells. Representative immunostaining with F4/80 mouse macrophage marker (data from *n* = 4 mice/genotype). Student’s *t*-test, *P < 0.05. Scale bar = 50 µm

To determine the relevance of our in vitro findings in vivo, we evaluated the macrophage infiltrate in a pituitary-specific *Aip*-knockout mice *Aip*^*Flox/Flox*^;*Hesx1*^*Cre/+*^ who develop GH-secreting pituitary tumors with disruption of the reticulin network (detailed description of this animal model will be reported separately). Based on our human data, we hypothesized that these animals will develop pituitary adenomas with significant macrophage infiltration. Immunohistochemical analysis with F4/80 macrophage marker from 15 months old homozygous *Aip*^*Flox/Flox*^;*Hesx1*^*Cre/+*^ mice pituitary glands showed a significant increase in the number of infiltrating macrophages as compared to age-matched wild-type mice (*P* < 0.05) (Fig. 6), similar to *AIP-*mutation-positive human samples. These results suggest that lack of AIP indeed leads to macrophage infiltration in pituitary tumors.

## Discussion

The tumor microenvironment plays a crucial role in the growth and invasion of tumors [7, 8, 10], but this has not been previously studied in aggressive pituitary tumors associated with *AIP* mutations. Using gene expression profiling of *AIP*pos human pituitary tumor samples, as well as in vitro and in vivo models, we established that *AIP*pos tumors have a unique microenvironment strikingly different from that of sporadic pituitary tumors. We identified increased number of tumor-associated macrophages in *AIP*pos tumors compared to sporadic ones. Similar to human *AIP*pos tumors, pituitary tumors from *Aip*-knockout animals exhibit increased macrophage content, suggesting that lack of *AIP* may be an important part of the molecular pathway leading to macrophage migration in both mouse and human pituitary tumors. Tumor-associated macrophages, typically with characteristics of activated macrophages, are an important component of the tumor microenvironment [22, 23] and correlate with poor prognosis in other tumor types [24]. Direct interactions between macrophages and tumor cells have been documented by multiphoton imaging [15, 25], and macrophages support tumor cell migration and invasion by secreting matrix degrading enzymes, such as plasminogen activator, cathepsin B and D, and matrix metalloproteases (MMP) 2 and 9 [26]. Indeed, *MMP2* and *MMP9*, known to be associated with cavernous sinus invasion [27], were upregulated in *AIP*pos samples (Table 1). Tumor-associated macrophages are linked with EMT [28, 29], which is present in AIP-deficient samples. MCM-treated GH3-*Aip*-KD cells undergo numerous changes associated with EMT, such as downregulation of E-cadherin and upregulation of ZEB1, remodeling of the cytoskeleton, and increased motility. However, there was no concomitant upregulation of classical mesenchymal markers, such as N-cadherin and vimentin [30–32] in our microarray data, suggesting a partial/incomplete EMT signature in *AIP*pos tumors. Partial or incomplete EMT has also been observed in other solid tumors [33]. Partial EMT was also found in some sporadic GH tumors [34], but the protein expression of AIP was not studied. As pituitary tumors locally invade but only very rarely metastasize, the partial EMT phenotype would match this clinical observation.

We saw increased expression of FOXP3+ T-reg cells in *AIP*pos tumors compared to sporadic adenomas and normal pituitary. FOXP3 is a specific T-reg marker that suppresses anti-tumor immune responses. FOXP3+ T-reg cells are associated with poor prognosis in various cancers [35, 36], and with EMT type tumor cells. Interestingly, CCL5, the cytokine we found overexpressed in *AIP*pos samples, recruited T-reg cells in a mouse model of pancreatic cancer [37]. Further studies will be needed to reveal the functional role of FOXP3 in pituitary tumors and to see whether CCL5 is indeed involved in recruitment of T-reg cells in *AIP*pos pituitary tumors.

Ingenuity pathway analysis of the differentially expressed genes of *AIP*pos, sporadic GH, and normal pituitaries highlighted the EMT pathway as one of the most significantly altered pathways in *AIP*pos tumors compared to sporadic adenomas. EMT is a highly conserved cellular process in which cells lose cell–cell contact and epithelial characteristics, and gain a motile and invasive mesenchymal phenotype, while mesenchymal-to-epithelial transition participates in the establishment and stabilization of distant metastases. In addition to their key role in development, EMT and mesenchymal-to-epithelial transition are involved with cancer progression. In *AIP*pos tumors we identified a significant number of altered EMT-associated genes, including epithelial markers (*CDH1*, *CTNNB1*, *ERSP1*, and *EPCAM*), a transcriptional (*ZEB1*) and a post-transcriptional regulator (*ESRP1*), while there were no statistically significant differences between sporadic adenomas and normal pituitaries. Therefore, significant disruption of the EMT pathway in *AIP*pos tumors may cause their more aggressive phenotype. Gene expression profiling and proteomics studies [38–46] led to the identification of genes associated with invasion and aggressive behavior [42, 47]. Changes in EMT markers have been seen in sporadic somatotroph adenomas with lower E-cadherin and ESRP1 expression [34, 48, 49]. Loss of ESRP1 in ~90% of *AIP*pos cases indicates that ESRP1 may be an important regulator of tumor invasiveness. GH itself has been suggested to stimulate EMT [50–52]: autocrine/paracrine GH or treatment with GH induces a complete EMT program and significantly up-regulates the classical mesenchymal markers such as N-cadherin and vimentin in some cancers [52–54]. Although high levels of GH raises the possibility that they play a role in the shift towards EMT in somatotroph tumors, not all somatotroph tumors show that EMT and EMT changes were not correlating with GH levels in sporadic somatotrophinomas [34, 48]. Comparison of our *AIP*pos tumor gene expression profile with that of *Aip*-knockout mouse embryonic fibroblasts [55] showed only a modest overlap. This could be explained by the different cell types as cAMP is stimulating cell proliferation in some cell types (e.g. adrenal and pituitary) while inhibits in others (e.g. fibroblasts and smooth muscle cells), and by the fact that AIP tumor suppressor role is specific to the pituitary gland.

While incubation with MCM leads to an EMT-like phenotype in both GH3-NT and GH3-*Aip*-KD cells, the degree of change is significantly different. Cell morphology parameters, EMT markers, and actin changes were more pronounced in GH3-*Aip*-KD cells, supporting the results on increased migration since in order for cells to invade through the extracellular matrix, filopodia/actin spikes protrude, which are crucial for successful migration/invasion. Media from macrophages stimulated GH3-*Aip*-KD cells to increase migration and invasion, while this cell type typically grown in complete medium do not show changes in migration/invasion assays [56, 57].

Next, we investigated the role of tumor-derived factors on macrophage recruitment. In the tumor microenvironment tumor cells interact with stromal cells either by cell–cell contacts or via paracrine signals. We hypothesized that tumor-derived chemokines might direct macrophage homing to the tumor microenvironment. We found increased expression of CCL5 in *AIP*pos tumors compared to the normal pituitary. Interestingly, our in vitro model confirmed these findings as GH3-*Aip*-KD cells secrete more than twice the amount of CCL5 into the media than GH3-NT cells. Elevated levels of CCL5 are associated with tumor progression in different cancers [58]. CCL5 is involved in the recruitment of monocytes, macrophages, and other inflammatory cells into inflammatory sites via activation of its receptors CCR1, CCR3, CCR4, and mainly CCR5. The CCL5/CCR5 axis plays an important role in the progression of a number of solid tumors (breast, ovarian, gastric, cervical, colorectal, and prostate) [59]. Maraviroc, a CCR5 antagonist initially approved for treatment of HIV infection, inhibits chemotaxis of macrophage and monocyte-derived dendritic cells towards CCL5 [60]. We demonstrated that CCL5-dependent chemotaxis significantly increased macrophage migration towards the GH3-*Aip*-KD-conditioned media compared to the GH3-NT-conditioned media and disruption of this signaling by maraviroc resulted in 50% reduction of macrophage migration. These results suggest that cells lacking AIP secrete a significant amount of CCL5, which can increase macrophage migration toward these cells and support macrophage migration into the tumor microenvironment, at least partly, by CCL5/CCR5-dependent chemotaxis. We also found upregulation of FLI1, the transcriptional regulator of CCL5, at the gene and protein level in *AIP*pos tumors [21]. Aberrant expression of FLI1 is associated with hematological malignancies and solid tumors [61–63]. Altered expression of FLI1 is also linked with tumor aggressiveness [63] and poor prognosis [64]. In our study, we have observed higher levels of FLI1 expression with the concomitant upregulation of CCL5 and the increased number of macrophages in human *AIP*pos tumors, supporting a crucial role for FLI1 and CCL5 in macrophage recruitment. Based on these data, CCL5/CCR5 appears to be a key factor in *AIP*-mutation-related tumorigenesis.

By identifying a novel regulatory pathway, our study raised further interesting questions. Functional links between AIP, FLI1, and CCL5 or mechanism/s of how AIP silencing stimulates FLI1 and subsequently CCL5 expression remain to be investigated. The role of AIP in immune-related process is interesting, since AIP is a co-chaperone of the aryl hydrocarbon receptor (AHR), a known immune regulator of T helper Th17 cells [65, 66]. Low level of AHR, which is found in *AIP*pos tumors [67], was found to be associated with EMT via autophagy, as the autophagy marker BNIP3 is inversely related to AHR protein levels [68]. Indeed, we observed a significant upregulation of *BNIP3* mRNA in *AIP*pos tumors.

Limitations of our study include the fact that we used a rat cell line as no human somatotroph pituitary cell line exists. The tumor microenvironment is complex of several cell types which, in addition to macrophages, might affect tumor cell behavior. Here we focused on macrophages, well known to be associated with EMT, but other cell types may also influence EMT in *AIP*pos tumors. We focused on pro-inflammatory cytokine CCL5 although our microarray data in human samples identified other significantly differentially expressed cytokines, such as TGFB, CCL4, and osteopontin, which will be explored in future studies.

In summary, our results using a unique resource of fresh frozen *AIP*pos tumors show an altered tumor microenvironment of *AIP*pos tumors compared to sporadic pituitary adenomas, where tumor-derived factors, such as CCL5, interact with macrophages resulting in increased infiltration, EMT, and more aggressive phenotype. Furthermore, as somatotroph tumors without *AIP* mutation can also exhibit low AIP protein expression, our findings could be relevant for a significant proportion of patients with somatotrophinomas. Immune infiltrates and EMT signatures might also be useful as biomarkers to stratify patient groups. Our results establish an important novel crosstalk between tumor cells and the surrounding tumor microenvironment and suggest potential targets for therapeutic interventions.

## Materials and methods

### Pituitary adenoma samples

Fresh frozen *AIP*pos growth hormone-secreting adenomas (*n* = 6) and sporadic GH-secreting adenoma (*n* = 4) (Table S5) were obtained at transsphenoidal surgery. A part of each sample was processed for routine histopathological and immunohistochemical studies, and a part was snap-frozen. Patients with sporadic tumors had no family history of pituitary or other endocrine tumors. Autopsy pituitary samples (*n* = 5) served as controls. For RT-qPCR validation all the 15 samples used for microarray analysis were included. For immunohistochemistry studies 8 additional *AIP*pos formalin-fixed paraffin-embedded tissue samples, as well as pituitary tissue microarray consisting of 34 sporadic somatotrophinomas and 13 normal pituitaries were used (Table S6).

### Gene expression analysis

Gene expression analysis was performed using Affymetrix Human Gene Chip HG-U133 Plus 2.0 array (Affymetrix, Santa Clara, CA, USA) (Supplementary material). Microarray data have been deposited to the National Centre for Biotechnology Information’s Gene Expression Omnibus (http://www.ncbi.nlm.nih.gov/geo, accession number GSE63357). Ingenuity Pathway Analysis, a web-based application (www.ingenuity.com), was used to analyze pathways and biological functions.

### Quantitative reverse transcriptase PCR

The gene-specific primer/probe sets for *CDH1*, *CTNNB1*, *ESRP1*, *PERP*, *EPCAM*, and *ZEB1* were purchased from Applied Biosystems (ABI, Foster City, CA, USA; Table S7). For details of RT-qPCR methods please see Supplementary Material.

### Protein detection

Immunohistochemical staining and immunoblotting was performed and scored as described in Supplementary Material using primary antibodies listed in Table S8.

### Cell line and in vitro functional study

We used rat pituitary cell line GH3 cells (obtained from European Collection of Authenticated Cell Cultures at the start of the project) and generated two stable knockdown cell lines, a 50% and an 80% knockdown, and a non-targeting control (GH3-NT) (Supplementary material). The 80% knockdown (GH3-*Aip*-KD) was used for the experiments unless otherwise stated. Cells were cultured in high glucose DMEM (Sigma, Gillingham, UK) supplemented with 10% heat-inactivated fetal bovine serum (FBS) and 1% penicillin and streptomycin. To collect GH3-conditioned media for ELISA measurement of CCL5 levels, GH3-*Aip*-KD and GH3-NT cells were seeded in six-well plates (2 × 10^6^), were grown for 24 h in 10% FBS DMEM and, following washing, incubated for 72 h in serum-free DMEM. To collect GH3-conditioned media for macrophage migration assay, cells (5 × 10^6^) were grown for 24 h in 10% FBS DMEM and, following washing, incubated for 72 h in serum-free DMEM for macrophage migration assays as chemoattractant. Functional assays were repeated three times and were performed at least in triplicate.

### Isolation and characterization of rat bone marrow-derived macrophages

Macrophages were isolated from rat bone marrow and cultures with granulocyte–macrophage colony-stimulating factor in RPMI with 10% FBS. The expression of macrophage markers CD11b and CD68 was assessed by immunofluorescence analysis (Supplementary material). At day 7 macrophages were treated with 320 nM PMA for 24 h and then media was replaced with 10% RPMI. After 72 h this media was collected and used as conditioned medium (MCM) for the subsequent analysis.

MTS cell proliferation and colony formation assays were performed as described previously [4]. Cell shape analysis, invasion assay, generation of *Aip* knockout mice, and statistical analysis are described in Supplementary material.

### Study approval

The study was approved by the Ethics Committee and patients gave written informed consent.

## Supplementary information

Supplementary materials

Supplemental Material 1

Supplemental Material 2

Supplemental Material 3

Supplemental Material 4

Supplemental Material 5
