# Supplementary material for: Tumor microenvironment defines the invasive phenotype of AIP-mutation-positive pituitary tumors
Source: Oncogene. 2019 Mar 12;38(27):5381–95. doi: 10.1038/s41388-019-0779-5 (PMC6755983; doi:10.1038/s41388-019-0779-5)
Supplement: Supplementary file 1 — Supplementary materials [file 41388_2019_779_MOESM1_ESM.docx]

**Supplementary material for:**

**Tumor microenvironment defines the invasive phenotype of *AIP*-mutation-positive**

**pituitary tumors**

Sayka Barry^1^, Eivind Carlsen^3^, Pedro Marques^1^, Craig E. Stiles^1^, Emanuela Gadaleta^2^, Dan M. Berney^2^, Federico Roncaroli^4^, Claude Chelala^2^, Antonia Solomou^1^, Maria Herincs^1^, Francisca Caimari^1^, Ashley B. Grossman^1^, Tatjana Crnogorac-Jurcevic^2^, Oliver Haworth^1^, Carles Gaston-Massuet^1^ and Márta Korbonits^1*^

**Corresponding author:**

Professor Márta Korbonits

Centre for Endocrinology, William Harvey Research Institute Queen Mary University of London,

Charterhouse Square, London EC1M 6BQ, UK

Phone: +44 20 7882 6238

Fax: +44 20 7882 6197

**Email:** m.korbonits@qmul.ac.uk­­­

**Supplementary Materials:**

**Patients and pituitary adenoma samples**

Clinical features and adenoma classification is shown in Table S5. Tumor size (microadenomas, <10mm and macroadenomas, >10mm in diameter) and invasiveness (with the signs of cavernous or sphenoid sinus invasion) were classified according to preoperative magnetic resonance imaging and surgical findings. Grade 1a (non-invasive tumor), Grade 1b (non-invasive and proliferative tumor) and grade 2a (invasive tumor), grade 2b (invasive and proliferative tumor) and grade 3 (metastatic tumor)^5^.

**Affymetrix Microarray analysis**

Total RNA was isolated from tissues using the RNeasy micro kit (Qiagen, Manchester, UK) according to the manufacturer’s protocol. RNA samples were assessed by a NanoDrop ND-1000 spectrophotometer (NanoDrop Technologies, Rockland, DE, USA) and further analysed by Agilent Bioanalyzer (Agilent Technologies, Palo Alto, CA, USA). Target labelling and hybridization were performed using Affymetrix GeneChip 3′ IVT Express Kit (Affymetrix, Santa Clara, CA, USA) according to the manufacturer’s instructions. Briefly, 250ng of total RNA was reverse-transcribed using the T7-(T)24 primer and cDNA synthesis kit. Double stranded cDNA was used as a template for *in vitro* transcription and amplification reaction in the presence of biotin-labelled ribonucleotides. Fifteen micrograms of labelled biotinylated cRNA was fragmented, mixed with the hybridization solution and hybridized to Affymetrix Human Gene Chip HG-U133 Plus 2.0 arrays for 16h at 45^o^C. Each array was washed and stained in a GeneChip Fluidics station 450 (Affymetrix) and scanned by a GeneChip 3000 scanner (Affymetrix) according to the manufacturer’s instructions.

PDAC TMA samples datasheet:

After hybridization and scanning, raw data files were analysed using Bioconductor packages (http://www.bioconductor.org) within the open source ‘R’ statistical environment (www.r-project.org). Quality control evaluations were conducted using the arrayMvout package in which nine quantitative features of array quality are assessed^1^. Any data files not passing these quality control checks were removed from subsequent analyses, with the remaining files being normalized using GC-Robust Multi-array Average^6^. The resulting normalized expression matrix was filtered using standard deviation calls to select the top 10,000 most variable probes across all experiments. To identify genes differentially expressed between the biological groups Bioconductor package limma was used^4^. Benjamini and Hochberg false discovery rate was applied for multiple testing corrections. A double cut-off of false discovery rate <0.05 and fold change of ≥2 was used. Microarray data have been deposited to the National Center for Biotechnology Information's Gene Expression Omnibus (http://www.ncbi.nlm.nih.gov/geo, accession number GSE63357).

**Ingenuity Pathway Analysis (IPA)**

Affymetrix probe identifiers and their corresponding ‘fold change’ values were uploaded to IPA and mapped to the corresponding object in the IPA Knowledge Base database. Canonical Pathway Analysis was performed. The significance values for Canonical Pathway Analysis is calculated using the right-tailed Fisher’s Exact test taking *P* value <0.05 being significant.

**Quantitative Reverse Transcription PCR (RT-qPCR)**

The gene-specific primer/probe sets for *CDH1*, *CTNNB1*, *ESRP1*, *PERP*, *EPCAM* and *ZEB1* were purchased from Applied Biosystems (ABI, Foster City, CA, USA; Table S7). Five hundred nanogram of total RNA from individual tissue samples were reverse transcribed into complementary DNA (cDNA) using random hexamers and the multiscribe reverse transcription kit (Applied Biosystems, Thermo Fisher Scientific). RT-qPCR reactions were prepared using Taqman Universal PCR Mastermix (Applied Biosystems, Thermo Fisher Scientific). The amount of target gene was calculated by interpolation from the standard curve after normalization to the endogenous control 18S according to the manufacturer's instructions (Applied Biosystems, Thermo Fisher Scientific). Normal pituitary was used as a calibrator and each of the normalized target values was divided by the calibrator normalized target value to generate the relative expression levels. The mean value of the normal tissues (5 pituitaries) was given a value of 1, and the mean value of the adenoma tissues was then expressed relative to the normal values.

**Immunohistochemistry**

Immunohistochemical staining was performed on 4µm paraffin-embedded tissue sections using the Ventana Discovery™ System, (Ventana, Illkirch, France). All slides were deparaffinised and processed for antigen retrieval with standard saline citrate buffer. After blocking, the slides were incubated with the respective primary antibodies (Table S8) and the appropriate horseradish peroxidase–conjugated secondary antibodies (Epitomics, Inc.,Burlingame, CA). A negative control was included with each run where the primary antibody was omitted. IHC was performed using the 3-3' diaminobenzidine (DAB) detection kit (Ventana) and the slides were counterstained with haematoxylin.

The stained tissue sections were scored by 2 pathologists blinded to the diagnosis of the sample on the basis of both the extent and the intensity of the immunoreactivity. The extent of immunoreactivity was scored according to the percentage of stained cells in relation to the entire section as (0 points for no staining, 1 point for less than 20%, 2 points for 20-50% and 3 points for more than 50% of the cells). The staining intensity was graded on a 0-3 scale 0 (no staining), 1 (weak immunoreactivity), 2 (moderate immunoreactivity) and 3 (strong immunoreactivity). The sum of the intensity and extent scores was used as the final staining score.

The numbers of CD68+ macrophages, CD8+, CD45RO+ and FOXP-3+ cells were then counted in three to five representative high power fields at x400 magnification depending on the size of the adenomas and averaged the scoring results.

**Generation of stable *Aip*-knockdown GH3 cells**

We have generated two lines of lentiviral-transduced shRNA knockdown of *Aip* (50% and 80%) in the rat pituitary somatomammotroph cell line GH3 and non-targeting shRNA controls (GH3-NT) in collaboration with Sirion Biotech. The cells show 80% reduced AIP protein were used in most experiments (GH3-*Aip*-KD) (Fig. S3C).

**Collection of GH3-*Aip*-KD cells conditioned media for CCL5 ELISA (Enzyme-Linked Immunosorbent Assay) analysis**

CCL5 protein was measured in conditioned media from GH3-*Aip*-KD cells using Mouse/Rat CCL5/RANTES Quantikine ELISA Kit (R&D Systems, Minneapolis, MN, USA). Cells (2x10^6^) were seeded in six-well plates, were grown for 24h and washed twice with serum-free DMEM and incubated with 1ml of serum-free DMEM. After 72h incubation, the medium was collected and centrifuged at 20,000 g for 15min at 4^o^C to remove cellular debris and the supernatants were assayed for CCL5 according to the manufacturer’s instructions. All experiments were performed three times in duplicates.

**Immunoblotting**

Cells (3x10^6^) were seeded in 10cm Petri dishes after overnight incubation cells were treated with MCM. After 72h cells were washed with PBS were harvested by scrapping and lysed in RIPA buffer. Whole cell lysates were incubated on ice 10min and then centrifuged at 20,000 g for 15min at 4^o^C. Protein concentration of the lysates was measured using Bradford protein assay (Bio-Rad, Hercules, CA). Thirty micrograms of total protein were separated by SDS-PAGE using NuPAGE® Novex® 4-12% Bis-Tris Gels (Abcam, Cambridge, UK) and transferred onto nitrocellulose membrane (GE Healthcare Life Sciences, Pittsburgh, PA). Membranes were blocked in 5% milk in TBST (Tris–Buffered Saline with Tween® 20) for 1h at room temperature and incubated with primary antibodies are listed in Table S8 and were detected with infrared fluorescent-labelled anti-rabbit or anti-mouse and anti-goat secondary antibodies at a 1:10,000 dilution using the Odyssey infrared-imaging system (Li-Cor, Lincoln, Nebraska USA). Densitometry was conducted on the resulting bands for both ‘housekeeping’ protein and protein of interest using Li-Cor software (Li-Cor, Lincoln, Nebraska USA).

**Migration assays**

Cell migration was evaluated by an in vitro wound-healing assay using Ibidi Culture-Inserts (two reservoirs; in µ-Dish 35 mm; Ibidi GmbH, Martinsried, Germany). Cells (70µl; 7×10^5^ cells/ml) were seeded in complete medium to the culture-inserts on top of a 6-well plate. After 24h of incubation at 37°C with 5% CO_2_, the culture inserts were removed to generate a cell-free gap of 500µm in a monolayer of cells. Detached cells were removed by replacing with the fresh media or macrophage conditioned media. Photographs of the gap area were taken immediately and then at the indicated time points by an inverted microscope at x4. Each experiment was repeated in triplicate. Images were analysed to measure the open wound area using the TScratch program (www.cse-lab.ethz.ch/software.html)^2^.

**Immunofluorescent immunostaining**

Cells (5x10^4^) were grown on 13-mm coverslips after 24h cells were fixed in 4% paraformaldehyde for 15min at room temperature, following washes in PBS cells were permeabilized (when necessary) with 0.1% Triton X-100 in PBS for 5 min at 4^o^C. Cells were then washed and blocked in 1% BSA for 30min at room temperature, then incubated with primary antibodies (listed in **Table S8**) followed by incubation with appropriate secondary antibodies (Alexa Fluor 568–conjugated goat anti-mouse IgG, Alexa Fluor 488–conjugated donkey anti-mouse IgG and Alexa Fluor 488–conjugated donkey anti-rabbit IgG; 1:1,000; Molecular Probes, Invitrogen) for 30 minutes. After stained with DAPI, 50 μg/mL 4',6-diamidino-2-phenylindole (DAPI; Molecular Probes) for 5min. The coverslips with the stained cells were then placed upside down on the glass slides using Permafluor aqueous mounting medium (Immunotech, Marseille, France) and left in the dark. Actin staining was performed using actin stain (Molecular Probes, 2 drops/ml). The stained slides were visualized and images were taken using a Zeiss LSM-510 confocal microscope at x63 magnification.

**Isolation and characterisation of macrophages**

Macrophages were isolated from rat bone marrow and cultured with GM-CSF in RPMI‑1640 medium (Invitrogen, Paisley, UK) with 10%, FBS and antibiotics. Briefly, the rat femur was isolated after removing all the muscle and sinew from the bone. The tips of the femur were cut and flushed out the bone marrow by inserting a 1ml syringe with a 22G needle filled with media in a 10cm petri dish under the hood. Using a 22G tipped syringe aspirated off the media containing the bone marrow into a 50ml Falcon tube and centrifuged (2000 rpm for 7min). Cells were counted and re-suspend in ~10^6^/ml of media with 1000U/ml recombinant rat GM-CSF (Peprotech, London, UK). Cells were plated in 10cm non-treated tissue culture plates. After 3 days, half of the media was changed with fresh media and cultured for 4 more days. On day 7, adherent cells were harvested with accutase solution (Sigma-Aldrich, MO, USA) and used for experiments.

**Collection of macrophage-conditioned media (MCM)**

Macrophages (at day 7 of differentiation) were treated with 320nM phorbol myristate acetate (PMA, Sigma) for 24h and then media was replaced with 10% RPMI. After 72h this media was collected and centrifuged and used as conditioned medium for the subsequent *in vitro* functional assays. The expression of macrophage markers CD11b and CD68 on these macrophages was assessed by immunofluorescence analysis.

**Macrophage migration assay towards GH3-*Aip*-KD cell-conditioned media**

To analyse the effect of GH3 cell-derived factors, macrophage migration assays were performed using 6.5mm transwell inserts with 8μm pores (BD Biosciences, CA, USA). PMA activated macrophages were pre-treated with 100nM maraviroc for 24h and seeded at 5x10^4^ cells (in 200μl of serum free medium) to the upper compartment and 750μl GH3-*Aip*-KD and GH3-NT conditioned media were added in the lower compartment. 100nM maraviroc was also added to the upper and lower compartment and incubated at 37°C and 5% CO2. After 24h medium and non-adherent cells were removed. Cells that had moved through the pores onto the lower surface of the filters were fixed with 100% methanol. After washing the membrane with dH_2_O, non-migrated cells at the top of the inserts are removed. The membranes were then stained with 2% Giemsa Blue (Sigma-Aldrich) and photographed at x10 magnifications using an Olympus CX41 microscope and the total number of migrating cells was counted in nine random fields. All assays were performed in triplicate and on three different days.

**Invasion assay**

Invasion assays were carried out using the BioCoat Matrigel Invasion Chambers with 8µm pores (24-well insert; BD Biosciences, CA, USA). Invasion Chambers are hydrated for 2h with 500µl of serum-free medium at 37^o^C. After rehydration of the Matrigel 750µl of fresh or macrophage conditioned media was added to the lower chamber as chemo-attractant and 2.5x10^4^ cells in 200µl serum-free medium was be added to the upper chambers and incubated for 72h at 37^o^C with 5% CO_2_. After 72h, the cells which invaded through the Matrigel membrane were fixed in 100% methanol and stained using 2% Giemsa blue (Sigma-Aldrich, MO, USA). The membranes were photographed in nine random fields at x10 magnification and the total number of invading cells of each chamber were counted and represented by the mean of three independent experiments.

**Cell shape analysis**

To quantify cell morphology six different cell shape parameters were measured by ImageJ software (version 1.36, National Institute of Health)^3^: *area* (area of selection in square pixels or in calibrated square units, μm^2^); *perimeter* (μm); *Feret’s diameter* (the longest distance between any two points along the selection boundary); *circularity* (4π × [Area][Perimeter]2 with a value of 1.0 indicating a perfect circle and close to 0.0, indicates elongated shape); *roundness* (4 × [Area] / π × [Major axis]^2^ the roundness is 1 for a circle and approaches 0.0 for very elongated shapes) and *solidity* ([Area] / [Convex area], describes the stiffness and deformability of an object, as the object becomes more solid, solidity value is 1.0). For each condition five images were taken at x40 for each time point for each experiment. On average 90-100 cells were analysed for each time point.

**Generation of *Aip*-knockout mice**

We have generated a novel mouse model with pituitary-specific bi-allelic *Aip* loss *via* crossing *Aip*^Flox/Flox^ (Jackson lab, New Jersey, USA) with *Hesx1*^Cre/+^(kind present from Prof Juan-Pedro Martinez-Barbera, University College London). The transgenic lines were kept on a C57/BL6 genetic background. Littermates not bearing the *Hesx1*^Cre^ allele were used as controls.

**Statistical analysis**

Statistical analyses were carried out using the PRISM statistical software package, version 6 (GraphPad, San Diego, CA). Student’s t-test, one-way or two-way ANOVA followed by Bonferroni or Neuman-Klaus posthoc tests and Kruskal-Wallis test followed by the Conover-Inman test were applied as appropriate. Data are presented as mean and standard error of mean (SEM), *P*<0.05 was considered significant.

**Supplementary Figures legends**

**Supplementary Fig. 1** T cells in human pituitary adenomas. Immunohistochemical analysis of CD8 (A) and CD45RO (B) with representative images in normal pituitary (NP, n=11), *AIP* mutation positive somatotroph adenomas (*AIP*pos GH, n=12) and sporadic somatotroph adenomas (Sp GH, n=17). Normal pituitary showed negative staining while *AIP*pos tumors and Sp GH showed some positivity but neither marker showed significance. All images are x200 magnification and scale bar=100µm.

**Supplementary Fig. 2** Ingenuity pathway analysis of canonical pathways significantly altered in *AIP*pos and sporadic somatotrophinomas. The differentially expressed genes in these two comparisons were analyzed using Ingenuity pathway multiple comparison analysis. The top significant canonical pathways are shown. The horizontal line parallel to the x-axis indicate a *P*=0.05 threshold. The ‘Regulation of the Epithelial-Mesenchymal Transition Pathway’ was one of the most significantly altered pathways in *AIP*pos GH tumors compared to sporadic adenomas (red arrow).

**Supplementary Fig. 3** Establishment of an *in vitro* model - GH3-macrophage co-culture. Macrophages were isolated from rat bone marrow and differentiated by GM-CSF (1000U/ml) (phase contrast images: first panel). On day 7, macrophages were treated without or with phorbol myristate acetate (PMA, 320nM) for 24h and characterized by immunofluorescence using macrophage markers (CD11b, CD68 and CD163) and actin (63x magnifications). PMA-activated macrophages showed more elongated morphology, expressed higher level of activated macrophage marker CD163. Scale bar=50µm. (B) Activated macrophages showed increased level of migration towards recombinant CCL5 compared to the unactivated macrophages and this was inhibited by maraviroc. (C) GH3-*Aip*-KD cells showing 80% reduction of AIP protein compared to the NT shRNA (n=5). GAPDH used as a loading control. (D) MTS cell proliferation and colony formation assays of GH3-NT and GH3-*Aip*-KD cells (with 50% and 80% reduction of AIP protein, respectively) showing a significant increase in cell proliferation at 48h and 72h time points as well as increased number of colonies in GH3-*Aip*-KD cells compared to the GH3-NT. *P* values indicated *, <0.05, **, <0.01 and ***, <0.001; two-way ANOVA and one-way ANOVA with Bonferroni multiple comparison tests, respectively. Data represent mean values of three independent experiments.

**Supplementary Fig. 4** MCM induced migration in GH3-NT and GH3-*Aip*-KD cells. Wound-healing assay representing migration at 0h and 6 days with or without MCM treatment. Wound-healing migration of GH3-*Aip*-KD cells increases following MCM treatment compared to the GH3-NT cells. Representative images of wound healing analysis showing percentage of closed area’ expressed as percentage of area covered/closed by the migrated cells are shown (x4), right panels. Scale bar=250µm.

**Supplementary Fig. 5** GH3-*Aip*-KD-derived conditioned media induces increased macrophage migration and show increased levels of secreted CCL5. (A) *In vitro* macrophage chemotaxis assay using GH3-*Aip*-KD and GH3-NT cell-derived medium as chemoattractant for migration of activated rat bone-marrow derived macrophages. Increased macrophage migration was observed towards the GH3-*Aip*-KD cell-derived medium compared to GH3-NT cell medium. (B) ELISA data showing significant increased levels of CCL5 in the GH3-*Aip*-KD conditioned media compared to GH3-NT conditioned media.

**List of Supplementary Tables**

**Table S1** Ingenuity pathway analysis – top canonical pathways (*AIP*pos, *AIP* mutation positive samples; Sp GH, sporadic somatotroph adenoma samples; NP, normal pituitary)

**Table S2** Validated six candidate EMT genes

**Table S3** Comparison of RT-qPCR and immunohistochemistry data for human pituitary tumor samples

**Table S4** Immunohistochemistry summary for six EMT genes

**Table S5** Clinical features of patients whose samples were used in the gene expression array

**Table S6** Additional sporadic GH and *AIP*pos cases used for IHC

**Table S7** Gene-specific primer/probe sets for RT-qPCR

**Table S8** Primary antibodies used for this study

| **Table S1** Ingenuity pathway analysis – top canonical pathways (*AIPpos*, AIP mutation positive samples; Sp GH, sporadic somatotroph adenoma samples; NP, normal pituitary) | | | | |
| --- | --- | --- | --- | --- |
| **Ingenuity Canonical Pathways** | **Analysis**  **Name** | **Number of molecules** | **log(*p-value*)** | **Molecules** |
| Molecular Mechanisms of Cancer | *AIPpos* vs NP | 83 | 3.34E00 | RAP2B,CDKN2A,GAB2,RAC2,JAK1,FZD3,SMAD3,HRAS,BMPR1B,MAPK13,CCND1,FGFR3,PAK1,CASP9,PIK3CG,MAP3K7,WNT4,IRS2,GSK3B,PRKD3,PRKD1,ITGA4,SMAD2,ARHGEF4,FGFR1,CDK6,RAC1,FGFR2,AURKA,APC,CDH1,MAX,CCND2,CDC42,ARHGEF16,PRKCH,FZD5,LEF1,CFLAR,FNBP1,CAMK2G,RELA,FYN,TCF4,PSENEN,BMP2,ARHGEF7,CTNNA1,GNA14,E2F3,PRKAG1,BRAF,FANCD2,BMPR1A,RHOD,PIK3C3,E2F5,RHOU,TGFB2,AKT3,BID,CTNNB1,CAMK2B,ITGB1,SRC,PRKDC,PMAIP1,ARHGEF12,GNAS,PIK3C2A,MAPK9,BAX,XIAP,ARHGEF5,RRAS2,PAK2,PIK3CB,BMP7,ADCY7,PRKAR1A,WNT5A,FZD7,CTNND1 |
| Molecular Mechanisms of Cancer | Sp GH vs NP | 34 | 3.86E-01 | BMP2,BMPR1B,GNA14,PRKAG1,FGFR3,E2F5,RHOU,AKT3,IRS2,WNT5B,ATM,ITGB1,PRKDC,ARHGEF4,PMAIP1,CDK7,FGFR1,CDK6,FGFR2,MAPK12,APC,PIK3R3,CCND2,RRAS2,PRKAG2,PIK3CB,FZD5,BMP7,CYCS,BMP6,GNAL,CDK2,FZD7,WNT5A |
| Leukocyte Extravasation Signaling | *AIPpos* vs NP | 49 | 2.81E00 | CD99,RAC2,CLDN11,CTNNA1,RAPGEF4,MAPK13,CLDN7,FGFR3,PIK3C3,PIK3CG,ARHGAP12,IRS2,ACTG2,CTNNB1,PRKD3,DLC1,PRKD1,ITGA4,TIMP2,ITGB1,SRC,PIK3C2A,FGFR1,RAC1,MAPK9,FGFR2,THY1,MMP2,NCF4,ITGB2,F11R,WIPF1,TIMP4,ARHGAP9,CLDN1,JAM3,RAP1GAP,ICAM3,CDC42,VAV3,NCF2,CD44,PECAM1,PIK3CB,PRKCH,CTTN,CLDN3,MMP9,CTNND1 |
| Regulation of the Epithelial-Mesenchymal Transition Pathway | *AIPpos* vs NP | 47 | 2.8E00 | LOX,RELA,ADAM17,TCF4,JAK1,PSENEN,FZD3,SMAD3,TWIST1,HRAS,FGF13,BRAF,FGFR3,NOTCH2,HGF,PIK3CG,PIK3C3,TGFB2,AKT3,WNT4,PERP, ESRP1, EPCAM, GSK3B,CTNNB1,EGFR,SMAD2,JAG2,ESRP2,PIK3C2A,FGFR1,FGFR2,MMP2,ZEB1,TCF7L1,APC,CDH1,CDH2,RRAS2,LEF1,FZD5,PIK3CB,MAP2K5,MMP9,CLDN3,FZD7,WNT5A |
| Regulation of the Epithelial-Mesenchymal Transition Pathway | Sp GH vs NP | 23 | 1.27E00 | ESRP2,FGF2,FGFR1,TWIST1,FGFR2,APC,FGF13,PIK3R3,FGFR3,NOTCH2,CDH2,FGF10,RRAS2,AKT3,IRS2,FZD5,PIK3CB,WNT5B,TCF7L2,EGFR,ATM,WNT5A,FZD7 |
| FAK Signaling | *AIPpos* vs NP | 27 | 2.62E00 | FYN,ARHGAP26,ARHGEF7,HRAS,TLN1,PTEN,FGFR3,PAK1,PIK3C3,PIK3CG,AKT3,IRS2,ACTG2,ITGA4,EGFR,ITGB1,SRC,PIK3C2A,ASAP1,FGFR1,RAC1,FGFR2,GIT2,DOCK1,RRAS2,PAK2,PIK3CB |
| FAK Signaling | Sp GH vs NP | 13 | 1.05E00 | ITGB1,PXN,ARHGAP26,FGFR1,FGFR2,PIK3R3,FGFR3,RRAS2,AKT3,IRS2,PIK3CB,EGFR,ATM |
| Paxillin Signaling | *AIPpos* vs NP | 29 | 2.46E00 | ARHGEF7,ITGA8,HRAS,TLN1,MAPK13,FGFR3,PAK1,ARFIP2,PIK3C3,PIK3CG,IRS2,ACTG2,ITGA4,ITGB1,SRC,PARVA,PIK3C2A,FGFR1,RAC1,MAPK9,FGFR2,GIT2,ITGB2,DOCK1,RRAS2,CDC42,PAK2,PIK3CB,ITGB6 |
| Paxillin Signaling | Sp GH vs NP | 14 | 9.79E-01 | ITGB1,PXN,PARVA,FGFR1,ITGA8,FGFR2,MAPK12,PIK3R3,FGFR3,RRAS2,IRS2,PIK3CB,ITGB6,ATM |
| Cdc42 Signaling | *AIPpos* vs NP | 31 | 2.39E00 | B2M,ARPC1B,ARPC5,HLA-B,EXOC6,MAPK13,EXOC7,CLIP1,HLA-G,PAK1,PPP1R12B,PPP1R12A,CFL2,BAIAP2,EXOC5,GSK3B,EXOC3,HLA-F,ITGA4,ITGB1,SRC,ACTR2,MAPK9,TNK2,APC,WIPF1,HLA-C,CDC42,PAK2,FCER1G,ARPC4 |
| Cdc42 Signaling | Sp GH vs NP | 16 | 1.17E00 | ITGB1,B2M,HLA-A,DIAPH3,HLA-B,EXOC6,MAPK12,APC,HLA-G,CDC42BPA,DIAPH1,PPP1R12B,CFL2,HLA-C,EXOC5,HLA-F |
| Remodeling of Epithelial Adherens Junctions | *AIPpos* vs NP | 19 | 2.38E00 | TUBB1,ACTR2,SRC,NME1,RAB5C,ARPC1B,MAPRE1,ARPC5,CTNNA1,CLIP1,APC,TUBB2B,CDH1,RAB5A,HGF,ACTG2,CTNNB1,ARPC4,CTNND1 |
| Fcγ Receptor-mediated Phagocytosis in Macrophages and Monocytes | *AIPpos* vs NP | 25 | 2.38E00 | FYN,GAB2,RAC2,ARPC1B,ARPC5,TLN1,PTEN,PAK1,PIK3CG,VAMP3,AKT3,ACTG2,PRKD3,FCGR3A/FCGR3B,PRKD1,SRC,ACTR2,RAC1,FYB,DOCK1,CDC42,SYK,VAV3,PRKCH,ARPC4 |
| Tight Junction Signaling | *AIPpos* vs NP | 39 | 2.24E00 | CPSF2,RELA,CLDN11,MARK2,CTNNA1,SYMPK,CLDN7,VTI1B,PRKAG1,PTEN,OCLN,PPM1L,TGFB2,VAMP3,CSTF2,AKT3,ACTG2,CTNNB1,CSTF1,TJP2,YKT6,RAC1,YBX3,CASK,CNKSR3,SNAP25,F11R,TJP3,JAM3,CLDN1,CDC42,NUDT21,PPP2R5E,PATJ,GOSR2,PPP2R1B,CSTF3,CLDN3,PRKAR1A |
| Tight Junction Signaling | Sp GH vs NP | 18 | 7.01E-01 | CPSF2,CLDN11,CSTF1,YKT6,TNFRSF1A,MARK2,MPP5,YBX3,CLDN7,CNKSR3,VTI1B,SNAP25,PRKAG1,TJP3,VAMP3,PRKAG2,AKT3,GOSR2 |
| Regulation of Actin-based Motility by Rho | *AIPpos* vs NP | 23 | 2.15E00 | ITGB1,ACTR2,RAC2,PFN1,ARPC1B,ARPC5,RAC1,PIP5K1B,GSN,PAK1,WIPF1,PPP1R12B,PPP1R12A,RHOD,CDC42,BAIAP2,PAK2,RHOU,ACTG2,PIP4K2A,FNBP1,ARPC4,ITGA4 |
| GM-CSF Signaling | *AIPpos* vs NP | 20 | 2.12E00 | PIK3C2A,FGFR1,RACK1,HRAS,FGFR2,CCND1,FGFR3,CSF2RB,RRAS2,PPP3R1,PIK3CG,PIK3C3,AKT3,IRS2,PIK3CB,STAT5B,STAT1,BCL2A1,CAMK2B,CAMK2G |
| GM-CSF Signaling | Sp GH vs NP | 10 | 9.98E-01 | FGFR3,PIK3R3,RRAS2,FGFR1,AKT3,FGFR2,IRS2,PIK3CB,STAT1,ATM |
| Signaling by Rho Family GTPases | *AIPpos* vs NP | 53 | 1.99E00 | RACK1,ARPC5,GNB5,PIP5K1B,CLIP1,FGFR3,PAK1,PPP1R12B,CFL2,PIK3CG,BAIAP2,IRS2,ACTG2,GNG12,ITGA4,ACTR2,SEPT8,ARHGEF4,NOX4,FGFR1,SEPT7,RAC1,FGFR2,CDH1,CDH2,CDC42,ARHGEF16,NCF2,PIP4K2A,FNBP1,RELA,ARPC1B,ARHGEF7,GNA14,CDH11,ARFIP2,PPP1R12A,CDH3,RHOD,PIK3C3,RHOU,ITGB1,ARHGEF12,GNAS,PIK3C2A,MAPK9,VIM,ARHGEF5,WIPF1,PAK2,PIK3CB,SEPT6,ARPC4 |
| Signaling by Rho Family GTPases | Sp GH vs NP | 27 | 8.8E-01 | CDH18,DIAPH3,GNB5,GNA14,CDH11,FGFR3,PPP1R12B,CFL2,EZR,RHOU,IRS2,GNG12,ATM,ITGB1,ARHGEF4,FGFR1,SEPT7,CDC42EP3,FGFR2,MAPK12,ROCK1,PIK3R3,CDH2,CDH8,PIK3CB,PIP4K2A,GNAL |
| Epithelial Adherens Junction Signaling | *AIPpos* vs NP | 33 | 1.88E00 | TCF4,ARPC1B,TGFBR3,ARPC5,CTNNA1,HRAS,CLIP1,TUBB2B,PTEN,NOTCH2,AGGF1,SORBS1,HGF,BAIAP2,TGFB2,AKT3,ACTG2,CTNNB1,EGFR,SRC,ACTR2,TUBB1,FGFR1,RAC1,TCF7L1,APC,CDH2,CDH1,RRAS2,CDC42,LEF1,ARPC4,CTNND1 |
| IL-8 Signaling | *AIPpos* vs NP | 43 | 1.79E00 | RAC2,RELA,RACK1,GNB5,HRAS,CCND1,EIF4EBP1,FGFR3,BRAF,RHOD,CXCR2,PIK3C3,PIK3CG,RHOU,AKT3,IRS2,PRKD3,PRKD1,GNG12,EGFR,SRC,NOX4,GNAS,PIK3C2A,FGFR1,RAC1,MAPK9,VEGFC,FGFR2,MMP2,BAX,IRAK3,ITGB2,ARRB2,CDH1,CCND2,RRAS2,NCF2,PAK2,PIK3CB,PRKCH,FNBP1,MMP9 |

| **Table S2** Validated EMT genes | | | | |
| --- | --- | --- | --- | --- |
| **Probe ID** | **Symbol** | **Name** | **Fold change** | ***P*-value** |
| 201131_s_at | *CDH1* | cadherin 1, type 1, E-cadherin (epithelial) | -27.00 | 0.00 |
| 223679_at | *CTNNB1* | catenin (cadherin-associated protein), beta 1 | -4.07 | 0.06 |
| 225846_at | *ESRP1* | epithelial splicing regulatory protein 1 | -32.33 | 0.00 |
| 201839_s_at | *EPCAM* | epithelial cell adhesion molecule | -2.42 | 0.04 |
| 222392_x_at | *PERP* | PERP, TP53 apoptosis effector | -3.73 | 0.03 |
| 210875_s_at | *ZEB1* | zinc finger E-box binding homeobox 1 | 3.64 | 0.01 |

| **Table S3** Comparison of RT-qPCR and immunohistochemistry data for human pituitary tumor samples | | | | |  |  |  |  |  |  |
| --- | --- | --- | --- | --- | --- | --- | --- | --- | --- | --- |
| **RT-qPCR** | | | | |  |  |  |  |  |  |
|  | **Overall *P* value** | **NP vs *AIP*pos** | **NP vs Sp GH** | ***AIP*pos vs Sp GH** |  |  |  |  |  |  |
| ***CDH1*** | *P* = 0.01 | significant | not significant | significant |  |  |  |  |  |  |
|  |  | *P* = 0.0047 | *P* = 0.2588 | *P* = 0.0017 |  |  |  |  |  |  |
| ***CTNNB1*** | *P* = 0.57 | not significant | not significant | not significant |  |  |  |  |  |  |
|  |  | *P* = 0.4544 | *P* = 0.3786 | *P* = 0.8098 |  |  |  |  |  |  |
| ***ESRP1*** | *P* = 0.01 | significant | significant | significant |  |  |  |  |  |  |
|  |  | *P* = 0.0059 | *P* = 0.0077 | *P* = 0.0001 |  |  |  |  |  |  |
| ***PERP*** | *P* = 0.03 | significant | not significant | significant |  |  |  |  |  |  |
|  |  | *P* = 0.0119 | *P* = 0.2476 | *P* = 0.0022 |  |  |  |  |  |  |
| ***ZEB1*** | *P* = 0.03 | significant | not significant | not significant |  |  |  |  |  |  |
|  |  | *P* = 0.0056 | *P* = 0.0997 | *P* = 0.1896 |  |  |  |  |  |  |
| ***EPCAM*** | *P* = 0.03 | significant | not significant | significant |  |  |  |  |  |  |
|  |  | *P* = 0.0114 | *P* = 0.5024 | *P* = 0.0043 |  |  |  |  |  |  |
| **Immunohistochemistry** | | | | |  |  |  |  |  |  |
|  | **Overall *P* value** | **NP vs *AIP*pos** | **NP vs Sp GH** | ***AIP*pos vs Sp GH** |  |  |  |  |  |  |
| **E-cadherin** | *P* = 0.002 | significant | not significant | significant |  |  |  |  |  |  |
|  |  | *P* = 0.0008 | *P* = 0.4359 | *P* = 0.001 |  |  |  |  |  |  |
| **Beta-catenin** | *P* = 0.03 | significant | not significant | significant |  |  |  |  |  |  |
|  |  | *P* = 0.0124 | *P* = 0.1922 | *P* = 0.0489 |  |  |  |  |  |  |
| **ESRP1** | *P* = 0.02 | significant | not significant | not significant |  |  |  |  |  |  |
|  |  | *P* = 0.0054 | *P* = 0.1288 | *P* = 0.136 |  |  |  |  |  |  |
| **PERP** | *P* = 0.05 | significant | not significant | significant |  |  |  |  |  |  |
|  |  | *P* = 0.0379 | *P* = 0.9893 | *P* = 0.0269 |  |  |  |  |  |  |
| **ZEB1** | *P* = 0.01 | significant | not significant | significant |  |  |  |  |  |  |
|  |  | *P* = 0.0062 | *P* = 0.5067 | *P* = 0.0184 |  |  |  |  |  |  |
| **CD68** | *P* = 0.0002 | significant | significant | significant |  |  |  |  |  |  |
|  |  | *P* = 0.0001 | *P* = 0.01 | *P* = 0.0005 |  |  |  |  |  |  |
| **FOXP3** | *P* = 0.009 | significant | not significant | significant |  |  |  |  |  |  |
|  |  | *P* = 0.01 | *P* = 0.2114 | *P* = 0.02 |  |  |  |  |  |  |
| **CCL5** | *P* = 0.0016 | significant | significant | not significant |  |  |  |  |  |  |
|  |  | *P* = 0.0011 | *P* = 0.0011 | *P* = 0.6075 |  |  |  |  |  |  |
| **FLI1** | *P* = 0.0064 | significant | not significant | significant |  |  |  |  |  |  |
|  |  | *P* = 0.0284 | *P* = 0.6159 | *P* = 0.0038 |  |  |  |  |  |  |

*P* values for multiple comparison analysis (Kruskal-Wallis test followed by the Conover-Inman test). (*AIP*pos, AIP mutation positive samples; Sp GH, sporadic somatotroph adenoma samples; NP, normal pituitary)

| **Table S4** Immunohistochemistry summary for EMT genes | | | |
| --- | --- | --- | --- |
| **Name** | **NP** | ***AIP*pos GH** | **Sporadic GH** |
| E-cadherin | 100% (12 of 12) positive | 84% (10 of 12) negative | 38% (13 of 34) negative |
| Beta-catenin | 100% (11 of 11) positive | 83% (10 of 12) negative | 40% (13 of 32) negative |
| ESRP1 | 64% (7 of 11) positive | 88% (8 of 9) negative | 50% (4 of 8) positive |
| PERP | 92% (12 of 13) positive | 83% (5 of 6) negative | 92% (23 of 25) positive |
| ZEB1 | 100% (11 of 11) negative | 73% (8 of 11) positive | 20% (3 of 15) positive |

| **Table S5** Clinical features of patients whose samples were used in the gene expression array | | | | | | | | | | | | | | | | |
| --- | --- | --- | --- | --- | --- | --- | --- | --- | --- | --- | --- | --- | --- | --- | --- | --- |
| ***AIP* mutation status** | **Gender** | **Diagnosis** | **Age at Diagnosis** | **Tumor** | **GH** | **PRL** | **ACTH** | **LH** | **FSH** | **TSH** | **Ki 67** | **Mitosis** | **Densely/Sparsely** | **Tumor grade^5^** | **Alive**  **Y/N** | **Disease**  **status** |
| *AIP*pos GH1 | F | Acromegaly | 44 | Macro | + | neg | neg | neg | neg | neg | 3% | 13/10 fields | Sparsely | 2b | N | Deceased |
| *AIP*pos GH2 | M | Gigantism | 17 | Macro | + | + | neg | neg | neg | neg | 6% | N/A | Sparsely | 2a | Y | Active |
| *AIP*pos GH3 | F | Acromegaly | 26 | Macro | + | + | neg | neg | neg | neg | <1% | no mitosis | Sparsely | 2a | Y | Active |
| *AIP*pos GH4 | M | Gigantism | 17 | Macro | + | + | neg | + | + | neg | 5-10% | N/A | Sparsely | 2a | Y | Cured |
| *AIP*pos GH5 | F | Acromegaly | 24 | Macro | + | + | + | neg | + | neg | 1-2% | N/A | Sparsely | 2a | Y | Active |
| *AIP*pos GH6 | F | Acromegaly | 27 | Macro | + | + | neg | neg | neg | neg | 5% | no mitosis | Sparsely | 2a | Y | Controlled |
| Sp GH1 | F | Acromegaly | 58 | Macro | + | neg | neg | neg | neg | neg | N/A | 1-1 mitosis | Densely | 1a | Y | Cured |
| Sp GH2 | M | Acromegaly | 40 | Macro | + | + | neg | neg | neg | neg | <1% | no mitosis | Densely | 1a | Y | Cured |
| Sp GH3 | F | Acromegaly | 61 | Macro | + | neg | neg | neg | neg | neg | neg | no mitosis | N/A | 1a | N/A | N/A |
| Sp GH4 | F | Acromegaly | 56 | Macro | + | occ+ | neg | neg | neg | neg | <1% | no mitosis | Sparsely | 1a | Y | Cured |
| NP1 | M | Normal pituitary | 62 |  | | | | | | | | | | | | |
| NP2 | M | Normal pituitary | 52 |  |  |  |  |  |  |  |  |  |  |  |  |  |
| NP3 | F | Normal pituitary | 35 |  |  |  |  |  |  |  |  |  |  |  |  |  |
| NP4 | M | Normal pituitary | 13 |  |  |  |  |  |  |  |  |  |  |  |  |  |
| NP5 | M | Normal pituitary | 23 |  |  |  |  |  |  |  |  |  |  |  |  |  |

N/A = not available; Y = yes; N = no

| **Table S6** Additional sporadic GH and *AIP*pos cases used for IHC | | | | | |
| --- | --- | --- | --- | --- | --- |
| **Type** | **Gender** | **Diagnosis** | **Age at Diagnosis** | **Tumor** | **Densely/Sparsely** |
| GH 5 | F | Acromegaly | 69 | Macro | Mixed |
| GH 6 | F | Acromegaly | 48 | Macro | Mixed |
| GH 7 | F | Acromegaly | 22 | Macro | Densely |
| GH 8 | F | Acromegaly | 46 | Macro | Densely |
| GH 9 | M | Acromegaly | 36 | Macro | Sparsely |
| GH 10 | M | Acromegaly | 36 | Macro | Sparsely |
| GH 11 | M | Acromegaly | 21 | Macro | Densely |
| GH 12 | M | Acromegaly | 33 | Macro | Densely |
| GH 13 | M | Acromegaly | 41 | Macro | Densely |
| GH 14 | M | Acromegaly | 50 | Macro | Mixed |
| GH 15 | F | Acromegaly | 31 | Macro | Densely |
| GH 16 | F | Acromegaly | 77 | Macro | Sparsely |
| GH 17 | F | Acromegaly | 51 | Macro | Densely |
| GH 18 | F | Acromegaly | 25 | Macro | Sparsely |
| GH 19 | M | Acromegaly | 32 | Macro | Densely |
| GH 20 | F | Acromegaly | 33 | Macro | Sparsely |
| GH 21 | N/A | Acromegaly | N/A | Macro | Densely |
| GH 22 | M | Acromegaly | 32 | Macro | Sparsely |
| GH 23 | N/A | Acromegaly | N/A | Macro | Sparsely |
| GH 24 | F | Acromegaly | 34 | Macro | Densely |
| GH 25 | F | Acromegaly | 25 | Macro | Mixed |
| GH 26 | M | Acromegaly | 42 | Macro | Sparsely |
| GH 27 | N/A | Acromegaly | N/A | Macro | Sparsely |
| *AIP*pos GH7 | M | Acromegaly | 29 | Macro | Sparsely |
| *AIP*pos GH8 | F | Acromegaly | 27 | Macro | Densely |
| *AIP*pos GH9 | M | Acromegaly | 35 | Macro | Sparsely |
| *AIP*pos GH10 | M | Acromegaly | 18 | Macro | Sparsely |
| *AIP*pos GH11 | M | Acromegaly | 24 | Macro | Sparsely |
| *AIP*pos GH12 | M | Acromegaly | 28 | Macro | N/A |
| *AIP*pos GH13 | F | Gigantism | 23 | Macro | Mixed |
| *AIP*pos GH14 | M | Gigantism | 20 | Macro | Sparsely |

| **Table S7** List of gene-specific primer/probe sets for RT-qPCR | |
| --- | --- |
| **Gene Symbol** | **TaqMan assay identity** |
| *CDH1* | Hs01023894_m1 |
| *CTNNB1* | Hs00355049_m1 |
| *ESRP1* | Hs00214472_m1 |
| *EPCAM* | Hs00901888_g1 |
| *PERP* | Hs00751717_s1 |
| *ZEB1* | Hs00611024_m1 |
| *18S* | Hs99999901_s1 |

| **Table S8** Primary antibodies used for this study | | | | | |
| --- | --- | --- | --- | --- | --- |
| **Antibody** | **Species** | **Company** | **Dilution IHC** | **Immunofluorescence** | **Western blot** |
| Actin green | Mouse | Molecular probes |  | 2 drops/ml |  |
| AIP | Mouse | Novus Biologicals |  |  | 1 in 1000 |
| Catenin beta-1 | Mouse | BD Transduction Laboratories™ | 1 in 50 |  |  |
| CCL5 | Mouse | PeproTech | 1 in 250 |  |  |
| CD11b | Mouse | Novus |  | 1 in 100 |  |
| CD163 | Mouse | AbD Serotec |  | 1 in 50 |  |
| CD45RO | Mouse | Dako | 1 in 100 |  |  |
| CD68 | Mouse | Dako | 1 in 200 | 1 in 50 |  |
| CD8 | Mouse | Dako | 1 in 100 |  |  |
| E-cadherin | Mouse | BD Biosciences | 1 in 50 | 1 in 50 | 1 in 100 |
| ESRP1 | Rabbit | Sigma | 1 in 50 |  |  |
| F4/F80 | Rat | Bio-Rad | 1 in 1000 |  |  |
| FLI1 | Rabbit | Thermoscientific | 1 in 50 |  |  |
| FOXP3 | Mouse | Abcam | 1 in 50 |  |  |
| GAPDH | Rabbit | Santa Cruz Biotechnology |  |  | 1 in 2000 |
| PERP | Goat | Abcam | 1 in 100 |  |  |
| ZEB1 | Rabbit | Santa Cruz Biotechnology | 1 in 50 | 1 in 50 | 1 in 100 |

**References**

1 Asare AL, Gao Z, Carey VJ, Wang R, Seyfert-Margolis V. Power enhancement via multivariate outlier testing with gene expression arrays. *Bioinformatics* 2009; **25:** 48-53.

2 Geback T, Schulz MM, Koumoutsakos P, Detmar M. TScratch: a novel and simple software tool for automated analysis of monolayer wound healing assays. *Biotechniques* 2009; **46:** 265-274.

3 Pasqualato A, Lei V, Cucina A, Dinicola S, D'Anselmi F, Proietti S *et al*. Shape in migration: quantitative image analysis of migrating chemoresistant HCT-8 colon cancer cells. *Cell Adh Migr* 2013; **7:** 450-459.

4 Smyth GK. Linear models and empirical bayes methods for assessing differential expression in microarray experiments. *Stat Appl Genet Mol Biol* 2004; **3:** Article3.

5 Trouillas J, Roy P, Sturm N, Dantony E, Cortet-Rudelli C, Viennet G *et al*. A new prognostic clinicopathological classification of pituitary adenomas: a multicentric case-control study of 410 patients with 8 years post-operative follow-up. *Acta Neuropathol* 2013; **126:** 123-135.

6 Wu Z, Irizarry RA. Preprocessing of oligonucleotide array data. *Nat Biotechnol* 2004; **22:** 656-658.
