# Supplementary figures and images for: Tumor microenvironment defines the invasive phenotype of AIP-mutation-positive pituitary tumors
Source: Oncogene. 2019 Mar 12;38(27):5381–95. doi: 10.1038/s41388-019-0779-5 (PMC6755983; doi:10.1038/s41388-019-0779-5)

SUPPLEMENTARY FIGURE 1

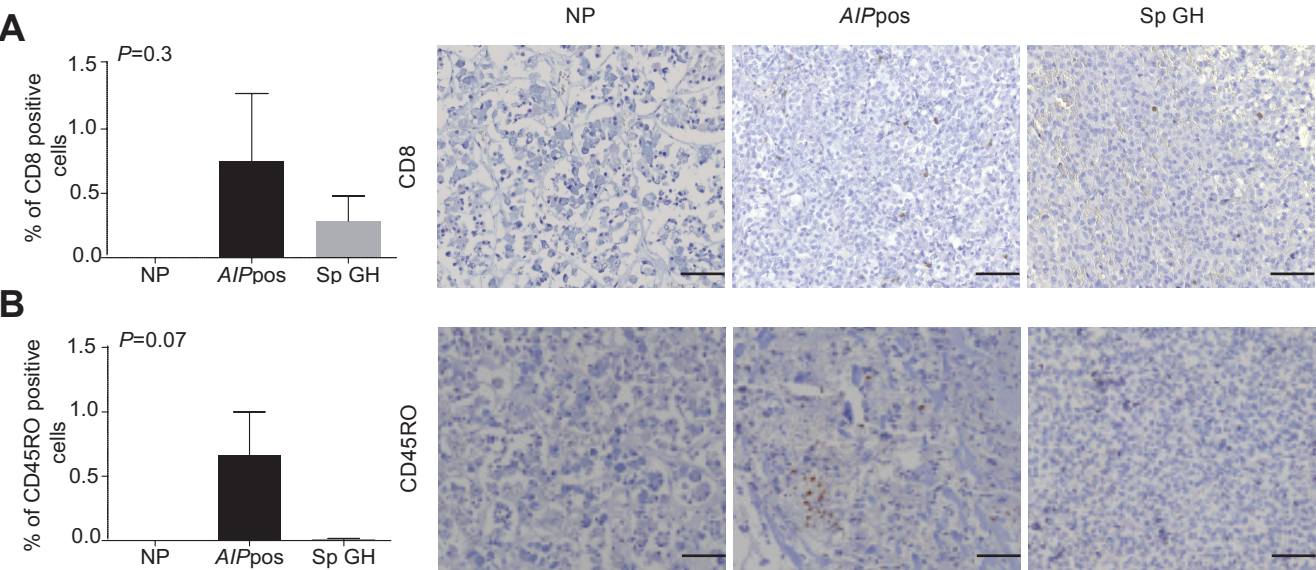

Supplement: Supplementary file 2 — Supplemental Material 1 [file 41388_2019_779_MOESM2_ESM.pdf]

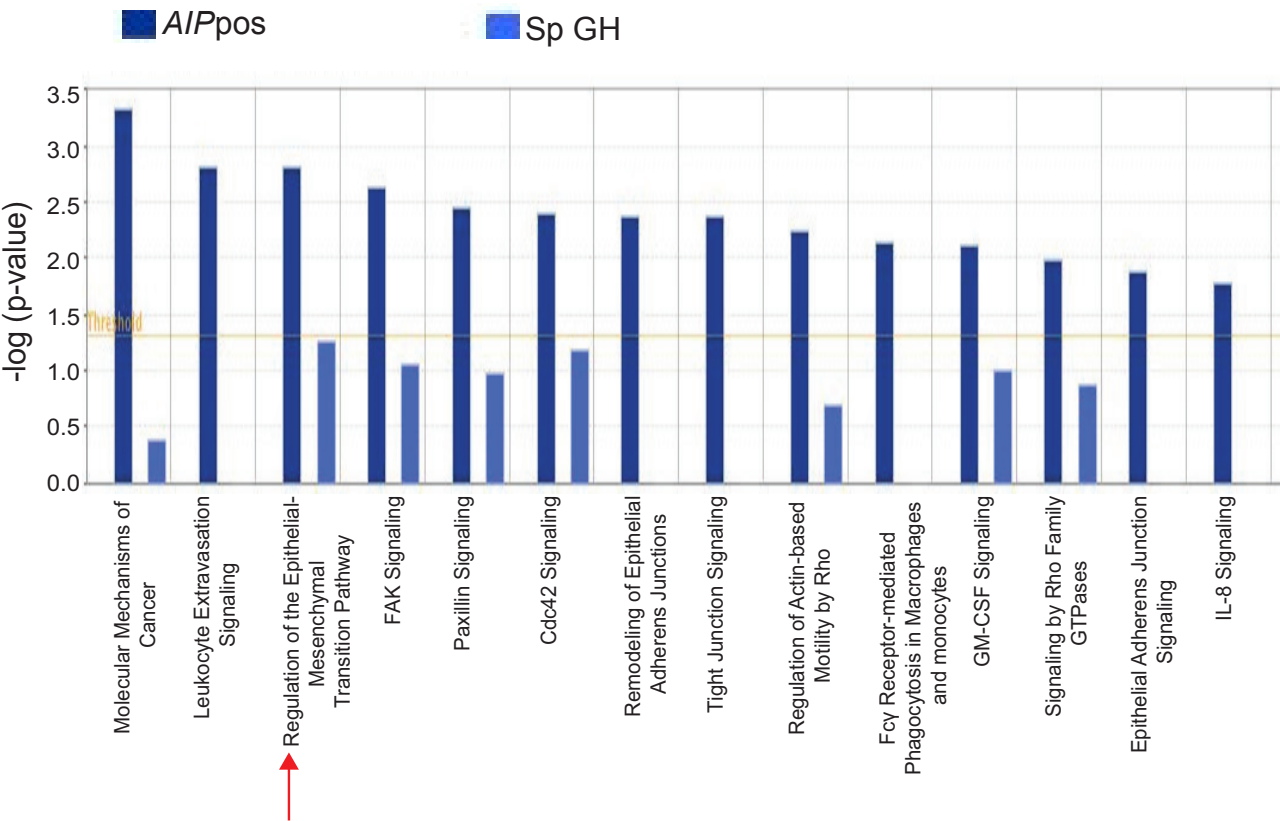

Supplement: Supplementary file 3 — Supplemental Material 2 [file 41388_2019_779_MOESM3_ESM.pdf]

SUPPLEMENTARY FIGURE 3

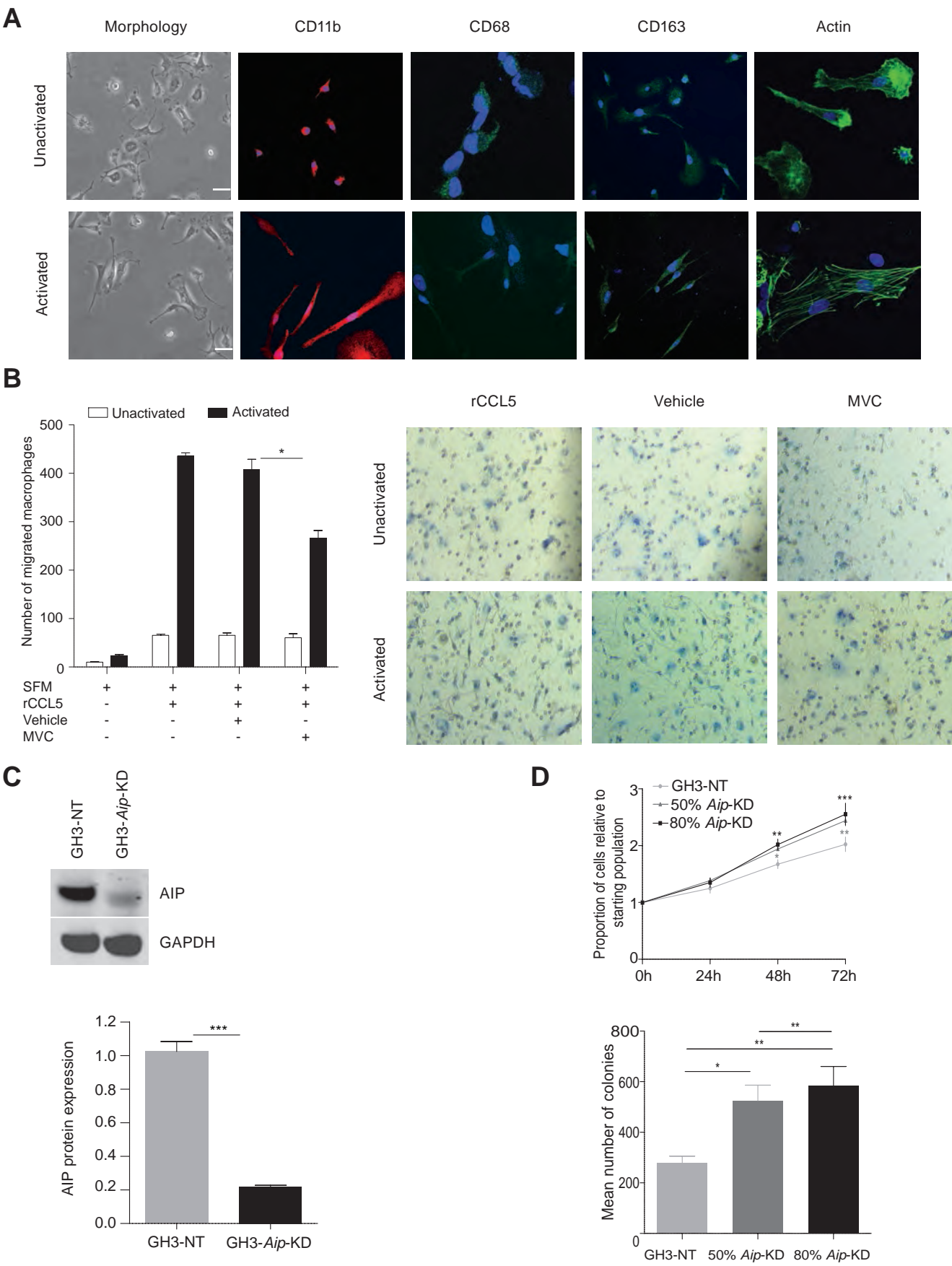

Supplement: Supplementary file 4 — Supplemental Material 3 [file 41388_2019_779_MOESM4_ESM.pdf]

SUPPLEMENTARY FIGURE 4

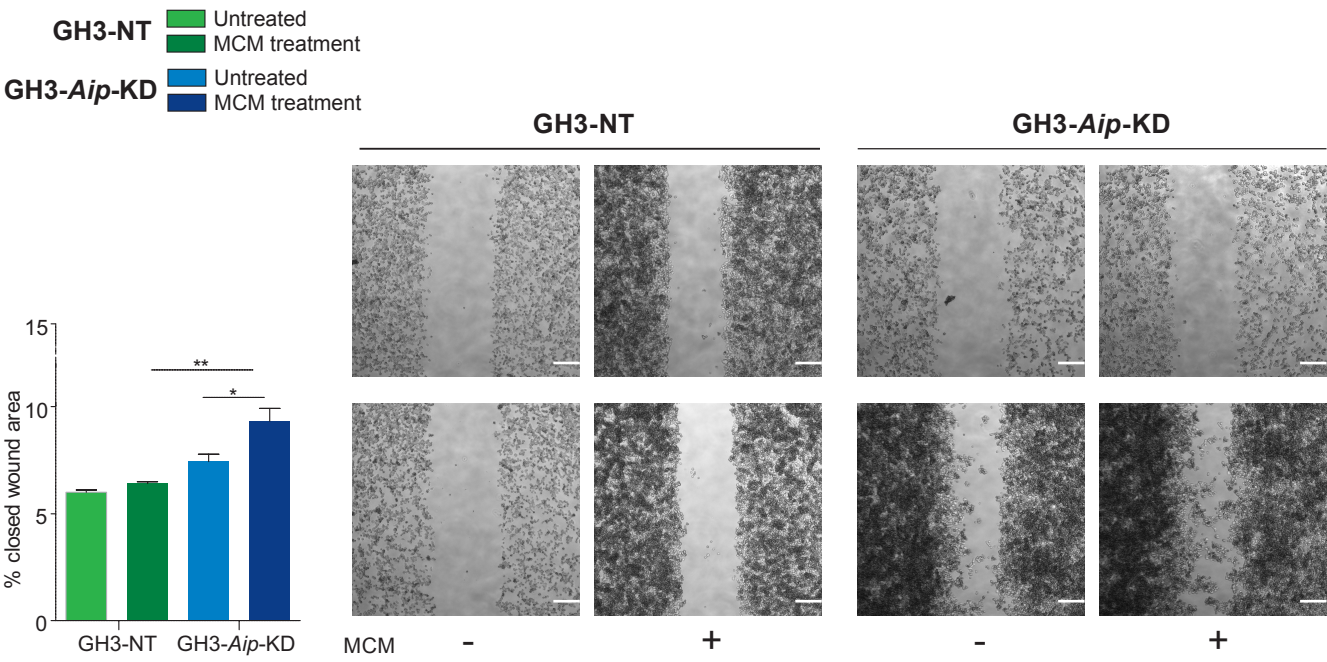

Supplement: Supplementary file 5 — Supplemental Material 4 [file 41388_2019_779_MOESM5_ESM.pdf]

**A**

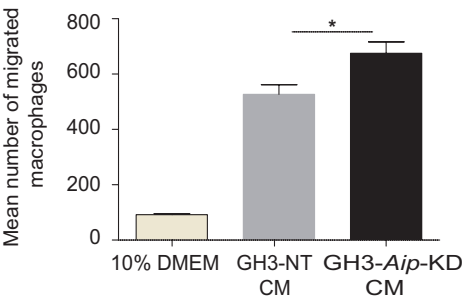

**B**

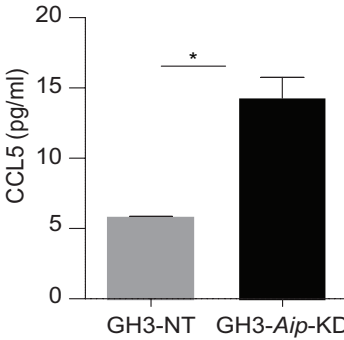

Supplement: Supplementary file 6 — Supplemental Material 5 [file 41388_2019_779_MOESM6_ESM.pdf]
